# Toward Chemotactic Supramolecular Nanoparticles: From Autonomous Surface Motion Following Specific Chemical Gradients to Multivalency-Controlled Disassembly

**DOI:** 10.1021/acsnano.1c05000

**Published:** 2021-09-22

**Authors:** Chiara Lionello, Andrea Gardin, Annalisa Cardellini, Davide Bochicchio, Manisha Shivrayan, Ann Fernandez, S. Thayumanavan, Giovanni M. Pavan

**Affiliations:** † Department of Applied Science and Technology, Politecnico di Torino, Corso Duca degli Abruzzi 24, 10129 Torino, Italy; ‡ Department of Innovative Technologies, University of Applied Sciences and Arts of Southern Switzerland, Polo Universitario Lugano, Campus Est, Via la Santa 1, 6962 Lugano-Viganello, Switzerland; § Department of Physics, Università degli studi di Genova, Via Dodecaneso 33, 16100 Genova, Italy; ∥ Department of Chemistry, Center for Bioactive Delivery at the Institute for Applied Life Sciences, University of Massachusetts, Amherst, Massachusetts 01003, United States

**Keywords:** chemotaxis, nanoparticles, stimuli-responsive, self-assembly, coarse-graining, molecular simulation, autonomous motion

## Abstract

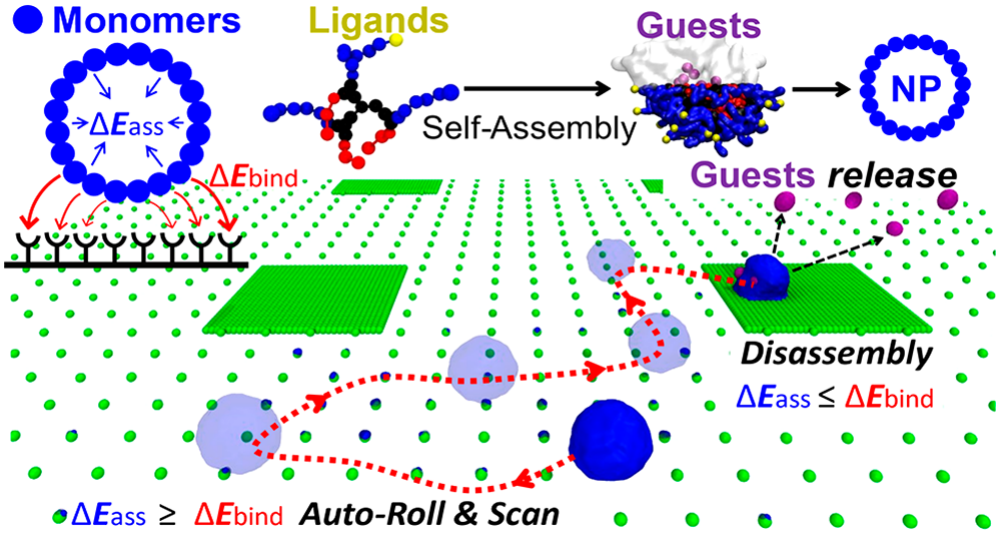

Nature designs chemotactic supramolecular structures that can selectively bind specific groups present on surfaces, autonomously scan them moving along density gradients, and react once a critical concentration is encountered. Since such properties are key in many biological functions, these also offer inspirations for designing artificial systems capable of similar bioinspired autonomous behaviors. One approach is to use soft molecular units that self-assemble in an aqueous solution generating nanoparticles (NPs) that display specific chemical groups on their surface, enabling multivalent interactions with complementarily functionalized surfaces. However, a first challenge is to explore the behavior of these assemblies at sufficiently high-resolution to gain insights on the molecular factors controlling their behaviors. Here, by coupling coarse-grained molecular models and advanced simulation approaches, we show that it is possible to study the (autonomous or driven) motion of self-assembled NPs on a receptor-grafted surface at submolecular resolution. As an example, we focus on self-assembled NPs composed of facially amphiphilic oligomers. We observe how tuning the multivalent interactions between the NP and the surface allows to control of the NP binding, its diffusion along chemical surface gradients, and ultimately, the NP reactivity at determined surface group densities. *In silico* experiments provide physical–chemical insights on key molecular features in the self-assembling units which determine the dynamic behavior and fate of the NPs on the surface: from adhesion, to diffusion, and disassembly. This offers a privileged point of view into the chemotactic properties of supramolecular assemblies, improving our knowledge on how to design new types of materials with bioinspired autonomous behaviors.

Nature offers numerous examples of supramolecular structures with fascinating dynamical chemotactic properties and stimuli-responsive behaviors.^1,2^ Cells, for example, can sense the density and distribution of extracellular matrix (ECM) molecules by means of surface proteins (integrins) and complexes.^1,2^ Such spatial sensing, based on the selective recognition/binding of ligands, controls and regulates the cellular activity in a variety of contexts.^3−5^ A specific example is offered by leukocytes, which recognize and react to surface markers indicative of an infection.^1,5−9^ In particular, leukocytes bind to the surfaces of blood capillaries, roll and scan surface markers, slow down, stop, and release inflammatory signals. Such binding, rolling, and reacting capabilities are controlled by a complex interplay between protein–protein and protein–carbohydrate interactions at the interface.^10−16^ While mimicking the complexity and autonomous fidelity of the immune system is a daunting challenge, imparting similar autonomous functionalities to synthetic materials (Figure 1a) would be a breakthrough in many fields, from biomedicine to sensing, and adaptive materials. However, addressing this challenge requires gaining fundamental insights on the molecular factors controlling the selective noncovalent interactions and the complex interplay (and competition) between them at the interface. Notable examples of synthetic supramolecular structures, such as fibers, vesicles, or tubes have demonstrated to have excellent stimuli responsive properties while autonomously moving.^6−12^ Also responsive nanoparticles (NPs) have shown surface binding capability combined with tailored releasing of encapsulated guests.^13−17^ To predict and engineer the selective binding on surfaces, both monovalent and multivalent affinities are exploited. To cite just a few examples, Liao *et al.* studied the correlation between monovalent labeling schemes on a gold NP and its diffusion rate on supported lipid bilayer membranes,^18^ while Overseem and co-workers investigated multivalent binding profiles of influenza virus on surfaces with receptor density gradients.^19^ However, despite notable advances in surface modification and control are emerging thanks to cutting-edge techniques,^20^ technical experimental limitations still prevent the rational design of chemotactic functional materials. First, tracking and observing the movement of soft, tiny NPs on surfaces at sufficiently small spatiotemporal scales is a hard challenge.^21,22^ Second, gaining insights on the molecular factors and processes that govern the NP chemotactic responsive behavior is even more complex, as it requires observing these materials in action at a submolecular resolution.

**Figure 1:**
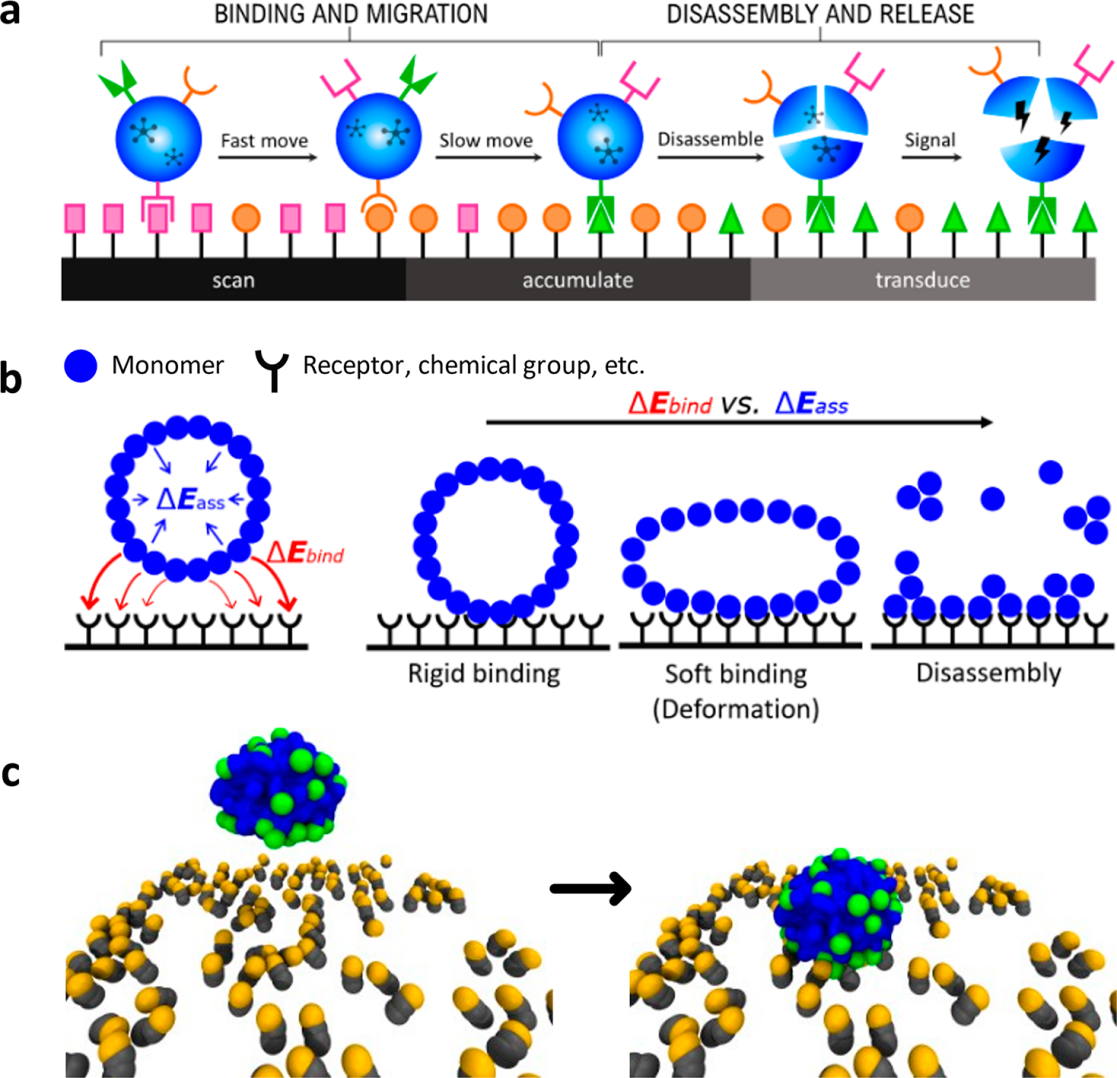
Multivalent adhesion and chemotaxis in natural and synthetic assemblies. (a) Cells (*e.g.*, leukocytes) can bind and autonomously roll/translocate on surfaces, scanning them and releasing (inflammatory) signals in the presence of high-densities of surface markers indicative of, for example, an infection. (b) Minimalistic coarse-grained (mCG) model of a self-assembled NP. When establishing a multivalent binding with a complementary functionalized surface, the NP’s fate depends on the competition between the monomers–receptors interactions (Δ*E*_bind_, in red) and the monomer–monomer self-assembly energy (Δ*E*_ass_, preserving the assembled structure, in blue). Whether the Δ*E*_ass_ globally prevails, competes with the Δ*E*_bind_, or is dominated by the latter, would result in a rigid, soft adhesion, or even in the disassembly of the NP. (c) Example of a molecular model of a supramolecular NP (mCG: monomers colored in blue and green) before (left) and after adhesion (right) on a ligand-coated surface (ligands in gray, active binding groups in orange).

Molecular models and computer simulations are fundamental to reach this goal. Recent simulations using minimalistic coarse-grained (CG) models allowed the study of the adhesion and dynamics of nanoparticles/cells (represented as single spheres) onto ligand-functionalized surfaces.^23−26^ These models permitted researchers to relate the number of interactions between the spherical nanoparticle (NP) and the surface receptors to the surface adhesion.^26,27^ Similar CG models also allowed simulation and monitoring of the rolling of a deformable (soft) spherical cell model on surfaces under the presence of an external flow.^24^ Recently, the diffusion profiles of a NP (modeled as a single sphere) on a fully cross-linked membrane CG model have been largely investigated.^27−29^ Specifically, variations of surface receptor density and multivalent interactions between the NPs and the gel-like membrane were observed to affect the diffusivity of the NPs, eventually inducing NP trapping in high-density regions.^27,29^ Although these interesting studies provided evidence of autonomous NP movement on surfaces, finer-level molecular models are needed in the perspective of designing supramolecular assemblies (*e.g.*, self-assembled NPs) which can selectively bind surfaces, autonomously scan them moving along chemical surface gradients, and trigger controlled dynamic responses (*e.g.*, NP binding, rolling, disassembly, and release of encapsulated guests). This requires (i) modeling the NP as an assembly of monomers (as the supramolecular structure of the NP must be explicitly taken into account in order to explore NP destabilization and disassembly) and (ii) keeping the resolution in the molecular models high enough to obtain chemically relevant insights into the molecular factors that control the behavior/fate of the NP on the surface. For example, it has been shown that ∼5 Å resolution CG models, coupled to advanced molecular simulations and analysis, allow the acquisition of precious links between the structure of monomers and the structure, dynamics, and dynamic properties of the supramolecular assemblies that these generate.^13,30,31^*In silico* simulations provided a privileged point of view into the response of supramolecular polymeric materials to different biorelevant stimuli, such as, for example, changes in temperature, salts, solvents, light, *etc*.^13,32^ All-atom molecular dynamics (AA-MD) simulations of protein-responsive assemblies allowed a comparison of the self-assembly stability of NPs composed of soft amphiphilic oligomers bearing biotin ligands (monomer–monomer interactions) with specific and nonspecific interactions with complementary extravidin. In particular, it was demonstrated how specific binding events with the complementary protein was capable of destabilizing the assembled NPs.^13^ However, it is worth noting that fascinating bioinspired properties such as chemotaxis have an intrinsically dynamic character. This encourages the study of the dynamic behavior of chemotactic assemblies at high (submolecular) resolution, in search of molecularly relevant information on how to control them.

Here we designed a reverse multiscale modeling approach to reach this goal. Starting from a minimalistic coarse-grained (mCG) model of supramolecular NPs which can selectively bind groups present on surfaces, we use classical and advanced simulation approaches to study their dynamic chemotactic behavior. Focusing on realistic example of supramolecular assemblies, we then increase the resolution of our models and investigate viable molecular ways to control the autonomous behavior of the responsive NPs on the surface. In the following we refer to this finer CG model using the acronym fCG. *In silico* experiments finally show us how to control the chemotactic properties and the dynamic disassembly of the supramolecular NPs. This multiscale approach offers a flexible platform toward the rational design of assembled structures with programmable autonomous chemotactic properties.

## Results and Discussion

### Chemotaxis of a Supramolecular Nanoparticle

Tracking and monitoring the dynamic behavior of soft assemblies on receptor-functionalized surfaces is crucial to understand how to design new types of artificial chemotactic NPs. We start from considering a supramolecular NP composed of self-assembling units (monomers) possessing ligands, or chemical groups, capable of establishing specific interactions with a complementary functionalized surface (Figure 1b). The fate of such a NP upon surface adhesion will essentially depend on the competition between the self-interactions of monomers in the assembly (Δ*E*_ass_) and the multivalent interactions (Δ*E*_bind_) with the surface. While the Δ*E*_ass_ governs the stability of the assembly, the Δ*E*_bind_ relates to the strength of specific interactions between the groups present on the monomers (*e.g.*, ligands, chemical groups, *etc*.) and the complementary ones on the surface (*e.g.*, receptors, complementary chemical groups). In fact, self-assembled polymeric NPs and micelles are far from behaving as rigid spheres. These are soft entities which may deform upon surface contact in the attempt of maximizing the interactions with the surface receptors by enlarging the contact area (Figure 1b). The interplay between Δ*E*_bind_ and Δ*E*_ass_ may produce different scenarios upon NP binding to the surface: (i) a rigid adhesion (for Δ*E*_ass_ ≫ Δ*E*_bind_, NP-surface binding has a negligible effect on the NP integrity), (ii) a soft adhesion accompanied by NP deformation (for Δ*E*_ass_ ∼ Δ*E*_bind_), or (iii) a potential destabilization and disassembly of the NP (for Δ*E*_ass_ ≪ Δ*E*_bind_). To challenge this simplistic scheme, herein we used coarse-grained (CG) molecular models (*e.g.*, Figure 1c).

**Figure 2:**
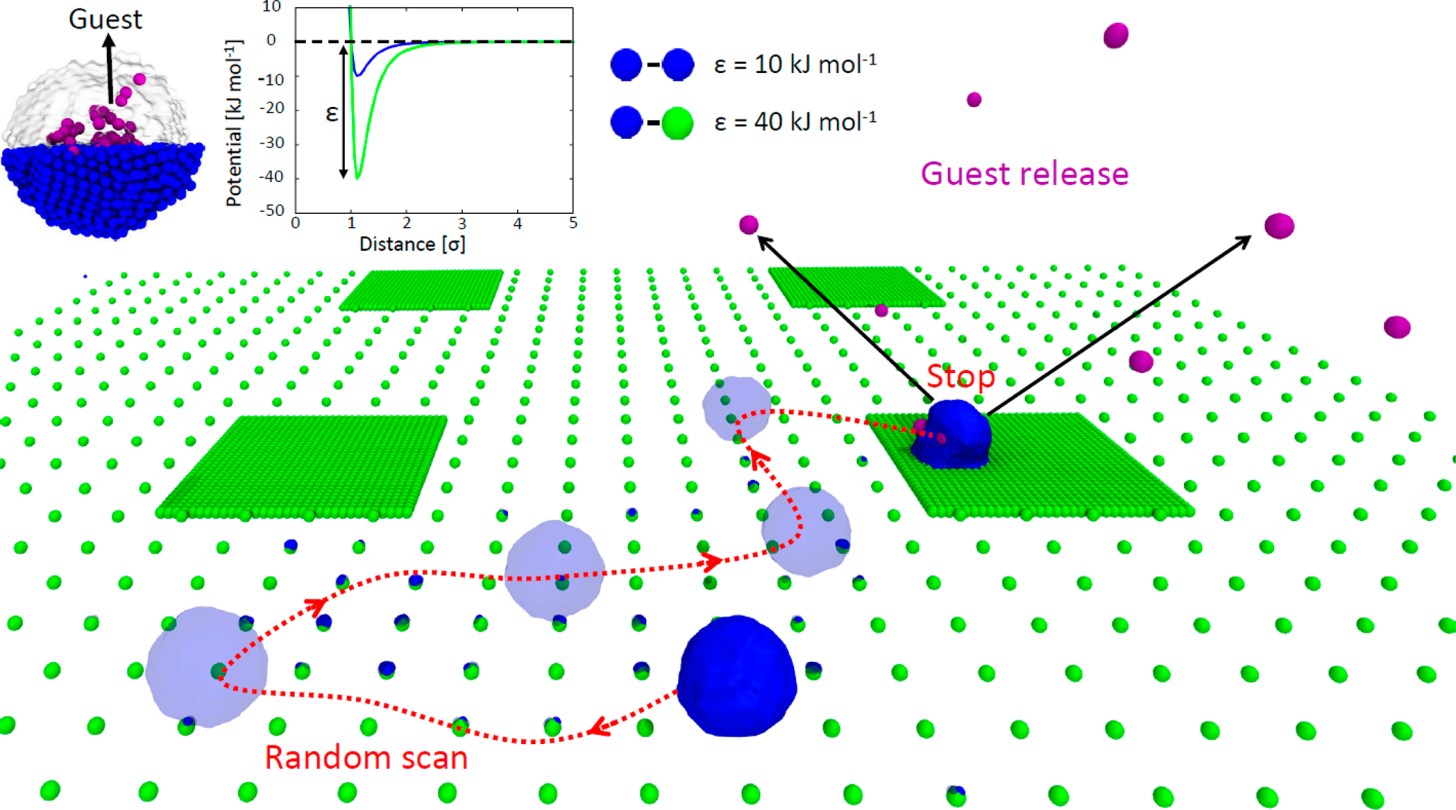
Simulating the density-responsive behavior of chemotactic NPs using minimalistic models. Minimalistic mCG model of a supramolecular NP (blue CG beads: self-assembled monomers) with encapsulated guests (violet CG beads). In the surface model, two areas are distinguishable: with high and low receptor densities (green CG beads). Top: the ε values of the 12–6 LJ potentials in this model are tuned to have a ratio between the strengths of the monomer–monomer and monomer-receptor interaction (Δ*E*_ass_/Δ*E*_bind_ ratio) of ∼1/4. Bottom: CG-MetaD trajectory (red dotted arrows) showing NP rolling and diffusion on the surface. Starting from a receptor-poor region, the NP randomly explores the surface during the CG-MetaD run, until reaching a receptor-rich region. Once the NP binds a receptor-rich region of the surface, the MetaD simulation suggests that the presence of a force continuously pulling the NP may induce NP disassembly over time, and the consequent release of the encapsulated guest particles (in violet).

We started developing a minimalistic, CG (mCG) model for a supramolecular NP composed of 1925 monomer units, each represented as a single CG particle (molecular resolution). To model the surface, we used a one-CG bead per-receptor group description, and we designed the surface in such a way to obtain two different (low and high) density areas on the surface. In the lower density region, the groups density is 1/64 than in the higher density region (Figure 2). The Δ*E*_bind_ and Δ*E*_ass_ interaction energies are modeled *via* Lennard-Jones (LJ) potentials (defined by LJ parameters σ and ε). Such a mCG model is approximated, and aims at providing general scope and qualitative insights. The interactions between the mCG particles in the model have been initially adjusted to obtain a Δ*E*_ass_/Δ*E*_bind_ ratio of ∼1:4 (similar to that recently estimated for self-assembling oligomers containing a biotin ligand able to specifically bind avidin).^13^

In this mCG model the monomer–monomer affinity, Δ*E*_ass_, is described by a 12–6 LJ potential using σ = 0.47 nm and ε = 10 kJ mol^–1^, while the monomer–receptor interaction, Δ*E*_bind_, is described by a 12–6 LJ potential using σ = 0.35 nm and ε = 40 kJ mol^–1^ parameters. On the entire surface, we added a weak 9–3 LJ attractive potential (2.0 kJ mol^–1^, considerably weaker than the specific interactions), which mimics weak nonspecific interactions between the NP and the surface in receptor-free surface regions. CG-MD simulations using such a simplified model show that the NP, even initially placed in proximity of the surface in the low receptor density region, tends to rapidly bind the surface, impeding the movement. Despite the rescaling of the specific Δ*E*_bind_ monomer–receptor interactions (set to 40 kJ mol^–1^ in this mCG model, while specific ligand–receptor interaction may be stronger; see the case of, for example, biotin–avidin binding, reaching ∼80 kJ mol^–1^),^13^ the characteristic time scales to escape the first NP–surface interactions exceed the typical time scales accessible by classical CG-MD simulations, which for this reason were found ineffective to study the dynamics of the system. Proven useful to study rare events in other complex supramolecular systems,^11,33^ we turned to an enhanced sampling approach, using metadynamics (MetaD) simulations to activate and explore the mechanism of motion of the NP on the surface. Depositing an energy bias on the *x* and *y* coordinates of the NP’s center of mass, CG-MetaD simulations allowed us to monitor the movement of the NP the surface. It is worth noting that such MetaD scheme biases only the position of the NP on the *xy* plane, while no constraint along *z* direction is imposed to the NP during the simulation. In this way, the biased simulation setup activates a random change of NP position on the *xy* plane, favoring a random walk on the surface where the NP can either slide, translate, roll, or in principle even bounce on/off the surface.

These CG-MetaD simulations show the NP moving in time from low to high density receptor regions on the surface, rolling in denser receptor regions and diffusing/sliding in the absence of receptors. No NP jumping was observed, highlighting how weak nonspecific interactions (in receptor-free regions) are enough to retain the NP in proximity of the surface. Although these CG-MetaD simulations have a purely explorative purpose (prohibitive convergence), these simulations provide a qualitative indication of the diffusion pathways of the NP on a receptor-functionalized surface. The observed diffusion of the NP on the surface is a combination of (i) the Brownian motion of the NP in a thermalized regime, and (ii) the specific interactions of the NP with the different group density regions of the surface. While (i) promotes the random movement of the NP on the *xy* plane, (ii) increases the residence time of the NP in surface regions richer in receptors (stronger and more stable binding). The NP is seen to move over time toward higher-density group regions during the MD simulations. This is the effect of the free energy of adhesion in regions of the surface where the density of receptors is different, which makes it less favorable/probable for the NP to escape from high-density rather than from low-density receptor regions. Moreover, it is worth noting that in the cases where the NP visits regions of the surface where the density of receptors is so high that Δ*E*_ass_ ≪ Δ*E*_bind_, on a sufficiently long time scale, for the NP it becomes more probable to disassemble rather than to escape from that region. Such high-density regions thus become density traps, which can even make irreversible the motion and the dynamic behavior of the NP on the surface.

As shown in Figure 2, two different behaviors of NP are evident: in low-density regions, the NP globally preserves its spherical shape during the diffusion. Individual monomers can be eventually lost during NP rolling, as the local receptor–monomer (receptor–ligand) interactions are stronger than the monomer–monomer interactions (see Supplementary Movie S1), but this does not perturb the integrity of the NP. On the other hand, in high-density-receptor regions, the NP tends to deform, due to the increased multivalent interactions with many receptors on the surface. Even after the NP reaches the denser receptors region on the surface, the MetaD scheme keeps pushing the NP to change its *xy* position, which, in the last part of the CG-MetaD run, results into a NP disassembly/exfoliation.

Similar to experimental setups, in these *in silico* experiments we also encapsulate guest CG beads (Figure 2, in violet) within the NP, which weakly interact with the other particles in the system (see Methods for details). Upon NP disassembly these are released in the surrounding environment (see Figure 2 and Supplementary Movie S1). It is worth noting that once the NP binds to the higher density region of the surface, the bias that keeps accumulating during the CG-MetaD run increases rapidly (see Supplementary Figure S4). This confirms that, in a realistic system, once the NP reaches a surface region with a high-density of receptors, the probability for NP escape from it (as an entire assembled entity) drops dramatically. Moreover, the NP behavior seen late during the CG-MetaD also qualitatively suggests that the presence of eventual external forces (or stimuli), which keep acting on the NP attempting to move it away from such stably bound configurations, may eventually induce the breakage of the NP and the consequent release of the encapsulated guests. Such an interesting hypothesis is tackled further in the next sections.

### Higher-Resolution Insights into the Effect of Multivalent Interactions

The preliminary evidence obtained through the minimalistic model (mCG model) of Figure 2 indicates that multivalent interactions (between the receptors on the surface and multiple ligands present on the NP) are key in controlling the chemotactic behavior of the NP on the surface. This suggests the intriguing perspective of controlling the autonomous behavior of the NP on the surface by rationally designing *a priori* the multivalent interaction between the self-assembled NP with the receptor-displaying surface. The mCG of Figure 2 offers a flexible platform to monitor, for example, the effect of monomer–monomer interactions (assembly stability), the influence of receptor density on the surface, or in general the impact of the relative strength of monomer–monomer *vs* monomer–surface interactions on the behavior of the NP. However, the molecular resolution of this minimalistic model (one particle per monomer) does not allow the acquisition of molecular-level information on how to practically control the NP chemotacticity. For example, it is known that a higher Δ*E*_ass_ would make the assembly more stable allowing in principle the entire NP to reach denser receptor regions. But what does this mean from a realistic, molecular point of view? How can one practically control Δ*E*_ass_, Δ*E*_bind_, and their ratio?

**Figure 3:**
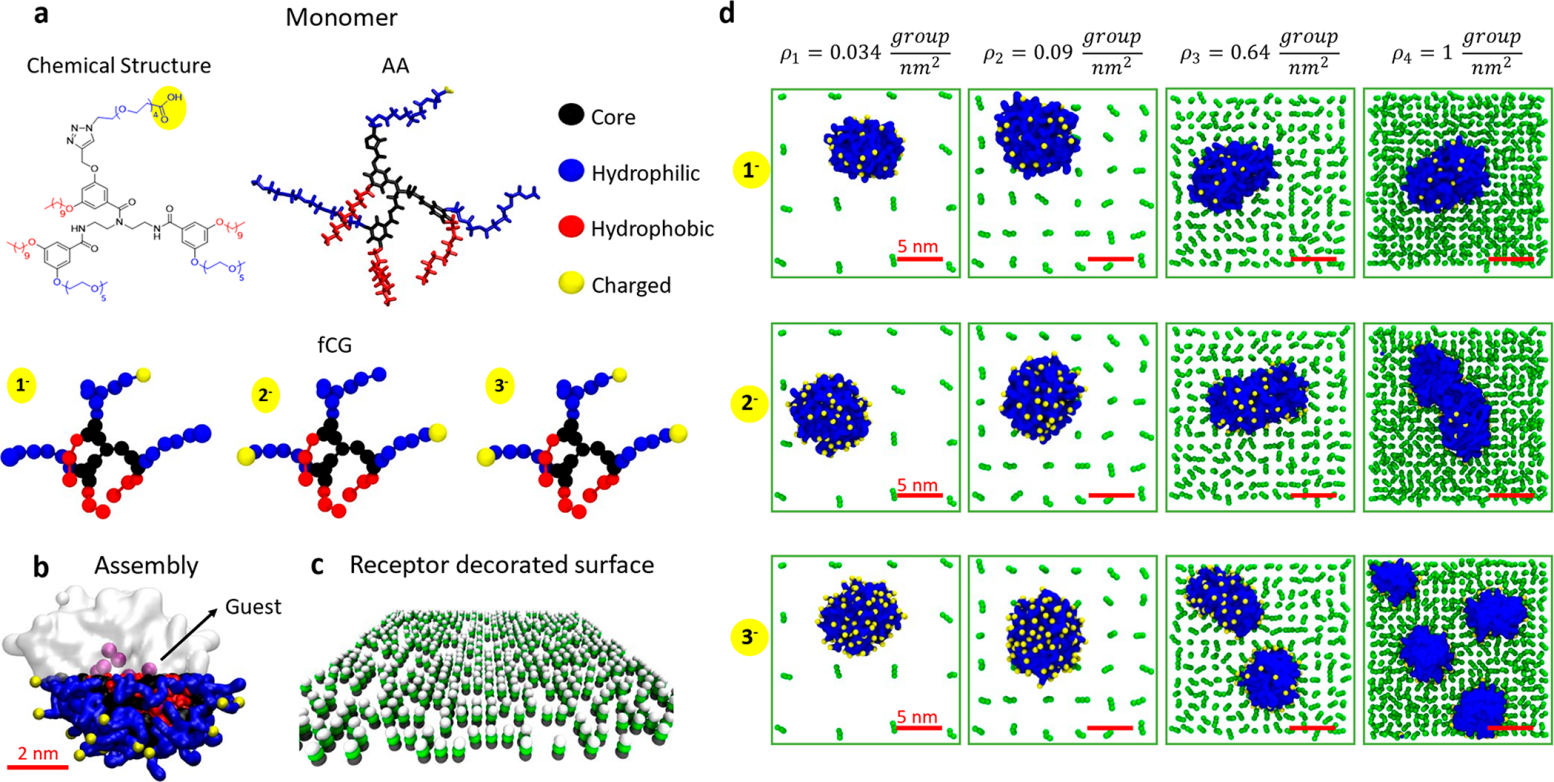
Submolecular resolution fCG models to study self-assembled NPs and their adhesion onto functionalized surfaces. (a) Chemical structure, all-atom (AA), and fine coarse-grained (fCG) models of facially amphiphilic modular oligomers. These self-assembling units are composed of a branched core (in black), hydrophobic groups (red) which trigger self-assembly in aqueous solution, and hydrophilic groups (red), which can be functionalized in different ways (*i.e.*, with COO^–^ charged groups, in yellow, in the example studied herein). (b) fCG model of a NP obtained *via* self-assembly of 44 oligomers in water. Guest fCG particles (in purple) are incapsulated spontaneously in the NP and used to monitor guest release upon eventual NP disassembly. (c) fCG model of a surface functionalized with +1*e* charged groups (dark green CG beads are constrained in their position, while the topmost white ones carry a +1*e* charge). (d) CG-MD simulation of static NP adhesion to surfaces characterized by different densities of receptor groups. Snapshots taken after 1 μs of fCG-MD showing NP destabilization and disassembly upon adhesion may be observed while increasing the charge densities on the surface and on the NP.

**Figure 4:**
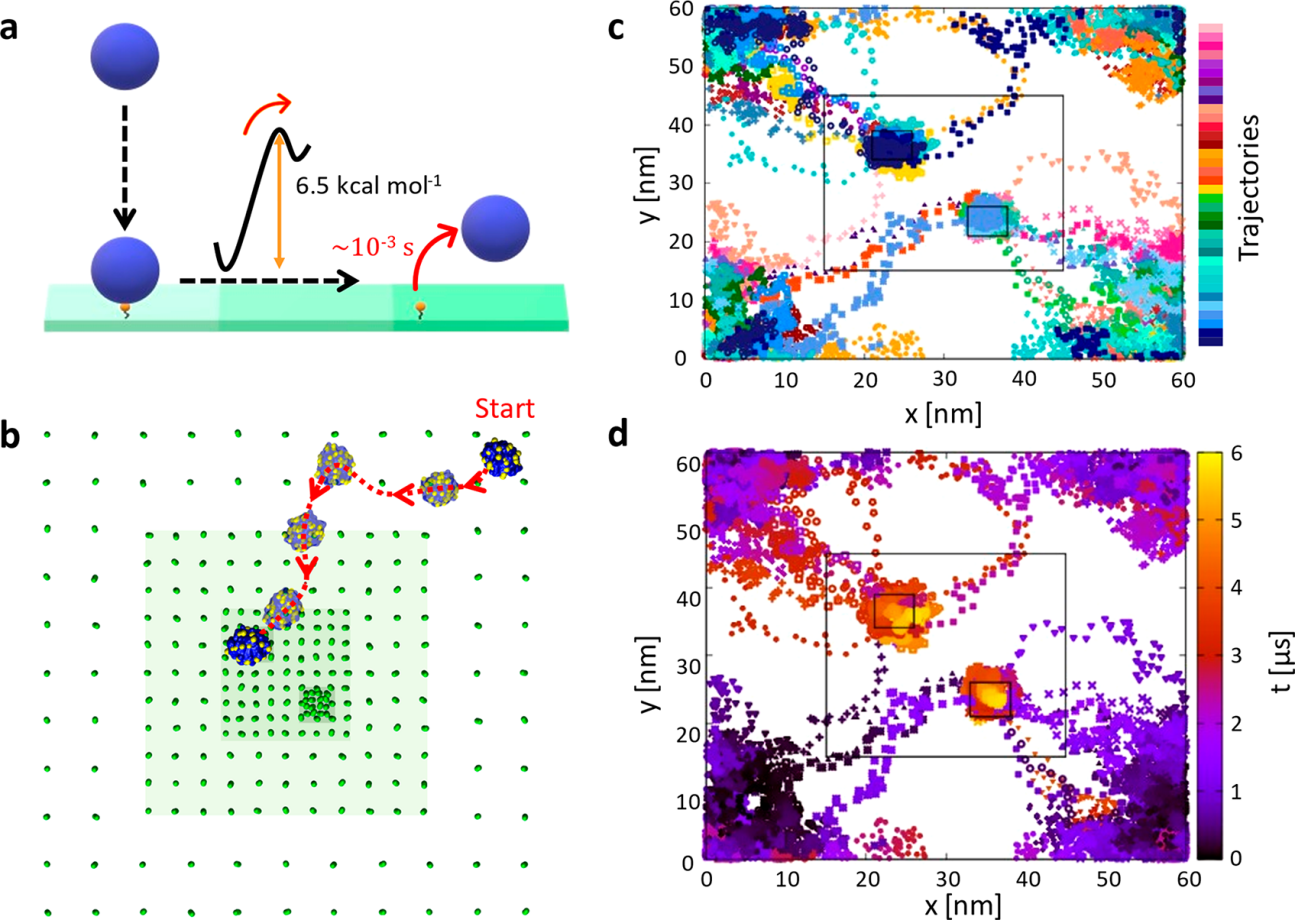
Submolecular fCG models of NP chemotaxis. (a) Free-energy barrier (∼6–7 kcal mol^–1^) and characteristic escape time scale (τ ∼10^–3^ s CG) for fCG-NP unbinding from the surface in the case of a monovalent interaction. (b) Example of CG-MetaD trajectory extracted from the ensemble of panels (c) and (d). (c) 36 trajectories of fCG-NPs on the surface from 36 multiple-walker CG-MetaD simulations (each color represents a different CG-MetaD run). (d) 36 multiple-walker CG-MetaD trajectories shown in panel (b), colored based on the simulation time (dark blue to red and yellow). In all CG-MetaD runs, the fCG-NP is seen to move from the lowest to highest-receptor density regions on the surface over time.

To answer such questions, a finer CG (fCG) model description is necessary. As a second step, we thus moved to fCG models of the system, where both the NP and the surface models are modeled with a ∼5 Å resolution. Higher resolution molecular models enable the study of the role of changing molecular structure of the self-assembling monomers, or for example, of the multivalent interactions between the monomers and the receptors present on the surface. However, while becoming more realistic molecularly (*i.e*., more chemically relevant), at the same time such submolecular resolution models become less general, as these have to refer to specific molecular structures.^34^ As an example, here we use as a reference case facially amphiphilic oligomers that self-assemble in aqueous solution forming NPs, which were recently demonstrated to allow successful encapsulation of hydrophobic guests in the NP interior.^13^ Thanks to their intrinsic multivalent modular nature, these self-assembling oligomer units are ideal platforms for this study. They possess hydrophobic and hydrophilic groups that can be individually functionalized and changed. This permits, for example, to graft onto their hydrophilic surface specific ligands (or chemical groups) that, exposed on the surface of the NP, allow the selective binding of determined receptors (or complementary chemical groups): changing the binding units and their number on the oligomer unit enables tuning of the Δ*E*_bind_. The use of such oligomers also permits modification of the hydrophobic groups,^35^ making the assembly more/less stable, changing the Δ*E*_ass_. Previous studies by our group demonstrated that molecular models can provide useful insights in such assemblies and in their stimuli-responsive behavior.^35−37^ In detail, the self-assembling oligomers that we employ here as a reference platform (Figure 3a) are composed of a branched scaffold, three hydrophobic decyl chains (hydrophobic face), and three hydrophilic polyethylene glycol moieties (hydrophilic face). Variable functionalities can be grafted onto the hydrophilic surface groups of these oligomers, which remain exposed on the NP surface upon oligomers self-assembly.^13,36^ In this case, we consider oligomers displaying a variable number of carboxylic acid functionalities (COOH groups). These are deprotonated at neutral pH (1, 2, and 3 COO^–^), imparting a charge of −1*e*, −2*e*, or −3*e* to the oligomers (Figure 3a). As seen in preliminary experimental evidence, this allows the acquisition of negatively charged self-assembled oligomer NPs (see Figures S1–S2), capable of binding to positively charged surfaces (Figure S3). First, we developed all atom (AA) models for these oligomers. We characterized (i) the behavior of a single oligomer in aqueous solution *via* AA-MD simulations. We also used AA-MetaD simulations to evaluate (ii) the oligomer–oligomer dimerization free-energy, estimating the strength of the interactions between two oligomers in solution (see Figure S5 in the Supporting Information). We then developed fine fCG models for the oligomers (resolution ∼5 Å, with a 3–4:1 heavy-atoms/CG-particle mapping), based on the widely used MARTINI force field scheme.^38^ In particular, given the spatiotemporal scales associated with the phenomena of interest herein, we considered the same protocol to develop and optimize an implicit-solvent version of these fCG models based on the dry-version of the MARTINI force field.^39^ First, we used the Swarm-CG software^40^ to optimize the bonded terms in the fCG model and hence to reproduce (i), second, the MARTINI bead types have been adjusted to obtain *via* CG-MetaD simulations dimerization free-energy profiles (ii) consistent with those obtained using the AA models (see Methods, and Figure S5).^30,41^ With this implicit-solvent fCG model, we obtained *via* self-assembly a NP model composed of 44 oligomers, spontaneously sequestering from the solution, and encapsulating 10 CG guest beads (Figure 3b, violet) during a CG-MD simulation. We also developed a model of a flat surface decorated with positively charged CG groups. The receptor groups are modeled as three CG beads, where the bottom one is constrained in its position, and the topmost one is +1*e* charged (Figure 3c). Four surface densities have been modeled (ρ_1_, ρ_2_, ρ_3_, ρ_4_), up to a maximum density of ρ_4_ = 1 charged-group/nm^2^ (Figure 3d), in the order of experimentally reported density values for amino-grafted surfaces.^42−45^ Complete details on the parametrization of the AA and fCG models are provided in the Methods section. We used these fCG models to study the NP adhesion on the surface. In particular, we were interested in observing the behavior of the NP following to the adhesion to the surface (in line to the hypothesized scheme of Figure 1b). CG-MD simulations of NPs composed of oligomers bearing 1, 2, or 3 COO^–^ charged groups (total NP charge of −44*e*, −88*e*, and −132*e*, respectively) binding surface models with growing densities of receptor groups (Figure 3d: from ρ_1_ to ρ_4_) clearly show that the behavior of the NP upon binding is strictly related to the strength of the multivalent NP–surface interactions, which depends on the density of charges present on the target surface, ρ, and on the NP (number of COO^–^ charged groups). Shown in Figure S6 (see Supporting Information), the NP adhesion to the surface increases moving from monovalent to trivalent self-assembled oligomers, as it is shown by the number of NP beads in contact with the surface receptor groups. Such evidence from the models also finds confirmation in structural illumination microscopy (SIM) experiments, showing an increment of signal related to surface bound NPs while increasing the multivalent interactions (see Figure S1 and Figure S2 of the Supporting Information). Noteworthy, the contacts between the oppositely charged groups of the NP and of the surface reach a maximum of ∼132 in the case of surface density ρ_4_ and trivalent NP oligomers, where complete NP disassembly can be observed during the CG-MD simulation (Figure 3d: bottom-right snapshot). While these results indicate that the strength of the NP-surface binding can be in general strengthened or weakened by playing either with the NP multivalent charges or with the surface distribution of receptor groups, unbiased CG-MD simulations were found ineffective to study the dynamic behavior of the NPs after surface binding (*e.g.*, in cases where the NP does not breakup upon adhesion). In particular, we used multiple infrequent CG-MetaD simulations to obtain qualitative information on the characteristic time scale for NP unbinding from a monovalent interaction with one positively charged surface group (see Methods for details). Analysis of the infrequent CG-MetaD simulations shows that the breakage of a monovalent interaction between one −1*e* charged group in the NP and one +1*e* charged group on the surface requires crossing a free-energy barrier of 6.5 kcal mol^–1^ (on average), with a characteristic escape/unbinding time estimated of ∼1.14 ms (∼10^11^ simulation timesteps, τ) at room temperature (see Figure 4a and Figure S8 in the Supporting Information). Transition times extracted from such fCG models should be considered as purely qualitative, and the variability and complexity of these systems makes it difficult to exactly reconstruct the kinetics for NP unbinding from spots where this establishes multivalent interactions with the surface. However, it is worth noting that, for the NP, escaping from stronger multivalent interactions with the surface can be only slower than escaping from monovalent interaction (this is consistent with preliminary experimental evidence showing system’s evolution in the time scale of minutes/hours). This indicates that the study of the dynamics of the NP after its binding to the surface far-exceeds the possibilities of classical MD simulations. To obtain an insight on the behavior of the system after the binding between the NP and the surface has occurred, we thus turned again to MetaD simulations. Accelerating the NP dynamics on the surface using a Gaussian-like bias potential on the *xy* position of the NP center of mass, allowed us to obtain qualitative insight on the diffusion behavior of the NP after binding to the surface. We carried out 36 multiple-walker CG-MetaD simulations, starting from a system configuration where the fCG-NP is placed in the lowest receptor-density region of the surface (*i.e*., on the corner of Figures 4b–d), and activating the fCG-NP exploration of the surface on the *xy* plane. During these runs, the fCG-NP can effectively explore different receptor-density regions of the surface (see also Supplementary Movie S2). This is consistent with preliminary microscopy experiments showing that movement after surface binding of NPs based on the same chemistry is possible (see Figure S3 in the Supporting Information). In particular, after a random movement around the initial NP position (corners of the simulation box), all 36 CG-MetaD simulations show that the fCG-NP tends to move from lower to higher density regions. From the temporal evolution of the fCG-NP displacement in 2D, we notice always a monodirectional motion of the NP, from the corner to the center of the surface model, despite the fact that the MetaD scheme used herein biases the random movement in 2D of the fCG-NP. The strong multivalent interactions established in the highest density areas makes it extremely unlikely for the NP to escape them and move further (Figure 4d: multivalent trapping of the NP).

To provide further evidence on this, we carried out two additional control CG-MetaD simulations (see Figure S7 in the Supporting Information), biasing the 2D fCG-NP motion onto simplified surface models. The results of such simulations confirm that these NPs indeed tend to move randomly in 2D as far as the surface density is uniform (the higher is the density of receptors, the slower is the 2D random motion). However, when, starting from low density regions, the fCG-NP moves onto a surface where the density of receptors is not uniform, it appears to move toward high density regions during the simulations, where it will keep moving randomly, albeit at a reduced speed. Such high receptor density areas are the surface spots onto which it will be statistically more likely to observe the NPs (as far as the condition Δ*E*_ass_ > Δ*E*_bind_ is respected), or to observe their disassembly on sufficiently long time scales (if Δ*E*_ass_ < Δ*E*_bind_).

It is interesting to note that in this finer fCG model, the NP-surface binding is mainly driven by electrostatic interactions, while in the minimalistic model (mCG) of Figure 2 this is controlled by van der Waals interactions (expressed by LJ potentials). Electrostatic interactions modeled *via* such simplified CG models should be handled with care, and the results obtained with these simulations have a purely qualitative purpose (see also Methods section). Nonetheless, it is worth noting that consistent NP behaviors are observed in both cases, independently of the type of interaction governing the specific NP–surface binding. This suggests that the autonomous migration of such chemotactic NPs following chemical surface gradients has a general character. In particular, rather than to the specific type of interaction, this again appears to be ascribable to the stronger/weaker multivalent interactions established by the NP in higher/lower density regions of the surface during its motion on the surface.

### Toward the Rational Design of Density-Responsive Chemotactic NPs

**Figure 5:**
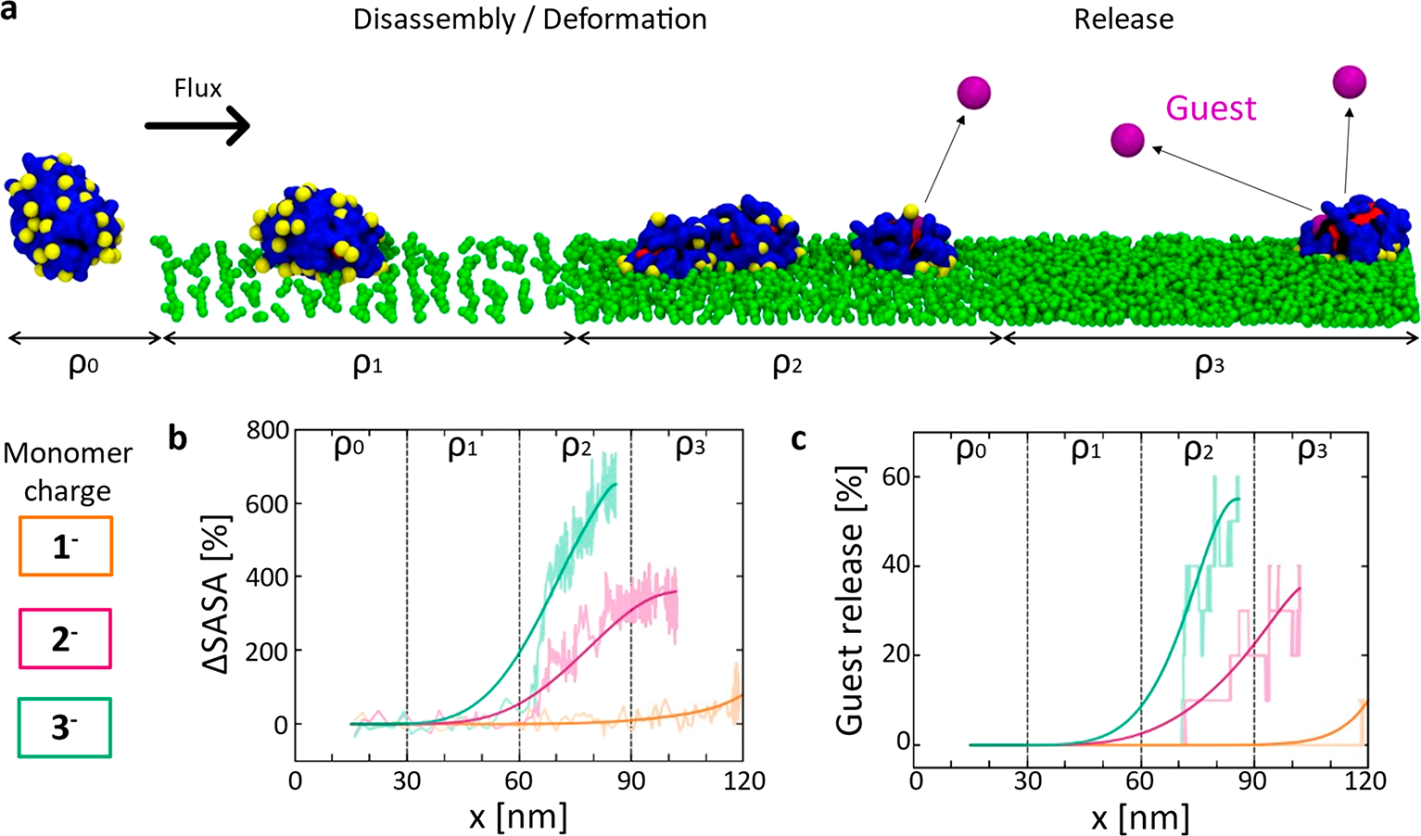
*In silico* experiments of NP rolling, disassembling, and guest releasing in the presence of an external flux. (a) CG-MD simulation setup of tested NPs represented with a fCG model. (b,c) Monitoring NP disassembly and guest release. (b) Percentage variation of the NP SASA (ΔSASA) for the NPs as a function of the oligomer charge. (c) Percentage of guest release as a function of the oligomer charge. Raw data are shown in transparent colors, solid trend lines are shown to guide the eye.

The previous sections suggest that it is possible, in principle, to design synthetic NPs which can follow chemical gradients on a surface, and stop once a determined density is met. In the perspective of mimicking the fascinating chemotactic properties seen in Nature, next questions are whether it is possible also to control the NP disassembly, and the release of the encapsulated guests, once a critical surface concentration is encountered, and eventually how. To challenge these points, we designed *in silico* experiments using our fine fCG models. In detail, we built a fCG model of a longitudinal surface functionalized with positively charged groups, the density of which grows along the main surface dimension (Figure 5a: from ρ_0_ to ρ_3_). One NP is initially placed in the ρ_0_, receptor-free region of the surface (Figure 5a, left). We then ran CG-MD simulations where a constant force is applied to the center of mass of the NP, continuously pushing the NP along the chemical surface gradient vector (*i.e*., from lower to higher density regions of +1*e* surface groups). The magnitude of the force was set in order to mimic the effect on the NP of an external flux comparable to that present in, for example, blood vessels (*i.e*., a NP directional diffusion rate of ∼0.5–1 cm/s, see Methods for details).^46^ While this is clearly a simplification of the effect of a realistic flux on the motion of the NPs, such an approximation is functional in our case. The main objective of these *in silico* experiments is, in fact, to obtain information on key molecular parameters that may allow to control the motion of such NPs on a receptor-density surface under perturbed conditions, similar to those present in realistic systems (*e.g.*, external flux, thermal agitation, *etc*.).

In some cases, we could observe that the NP assembly was pushed by the force until reaching a complete breakup and release of the encapsulated guests (Figure 5a), while in other cases the NP was seen to deform without disassembling during the surface rolling (see Supplementary Movies S3, S4, S5). NP disassembly and deformations were mainly monitored by evaluating the variations of the NP solvent accessible surface area (SASA) during the CG-MD run.

The results show that the NP tends to establish a higher number of multivalent interactions with the surface groups while moving toward denser surface regions, as recently demonstrated with fluorescent microscopy experiments in other multivalent binding systems.^19^ Such enhancement of the multivalent binding with the surface leads to an increased exposure of the hydrophobic parts of the NP oligomers to the solvent, thereby entailing an increase of the SASA of the assembly (ΔSASA). The ΔSASA increases even further as a consequence of NP exfoliation, as well illustrated in Figure 5b. Interestingly, when the number of charges on the oligomer surface is increased (*i.e*., enhancing the NP multivalency), the NP disassembly occurs earlier, at lower surface densities of receptor groups (Figure 5b). The number of contacts between the encapsulated guest fCG particles and the oligomers in the NP also provides indication on the stability of the guest encapsulation and of their eventual release. In particular, by calculating the extent of the drop in the guest–oligomer contacts during the CG-MD runs, we can estimate the percentage of guest release as a function of the surface density of receptors (ρ). As shown in Figure 5c, the guest release is associated with the NP disassembly. Our simulations clearly highlight that by modulating the number of charges on the oligomers it is possible to trigger the release of the guests, and in principle also to control at which receptor density this takes place. Given the statistical relevance of the results and the soft/dynamic character of these assemblies, percentages of release >20% in Figure 5c can be considered indicative of effectively releasing systems (even at the experimental level, a residual release of ∼10% is intrinsically present in such types of soft assemblies, independently of whether these disassemble or not),^13^ although we underline that the most relevant information in this sense is qualitatively obtained from the trends of the release profiles.

**Figure 6:**
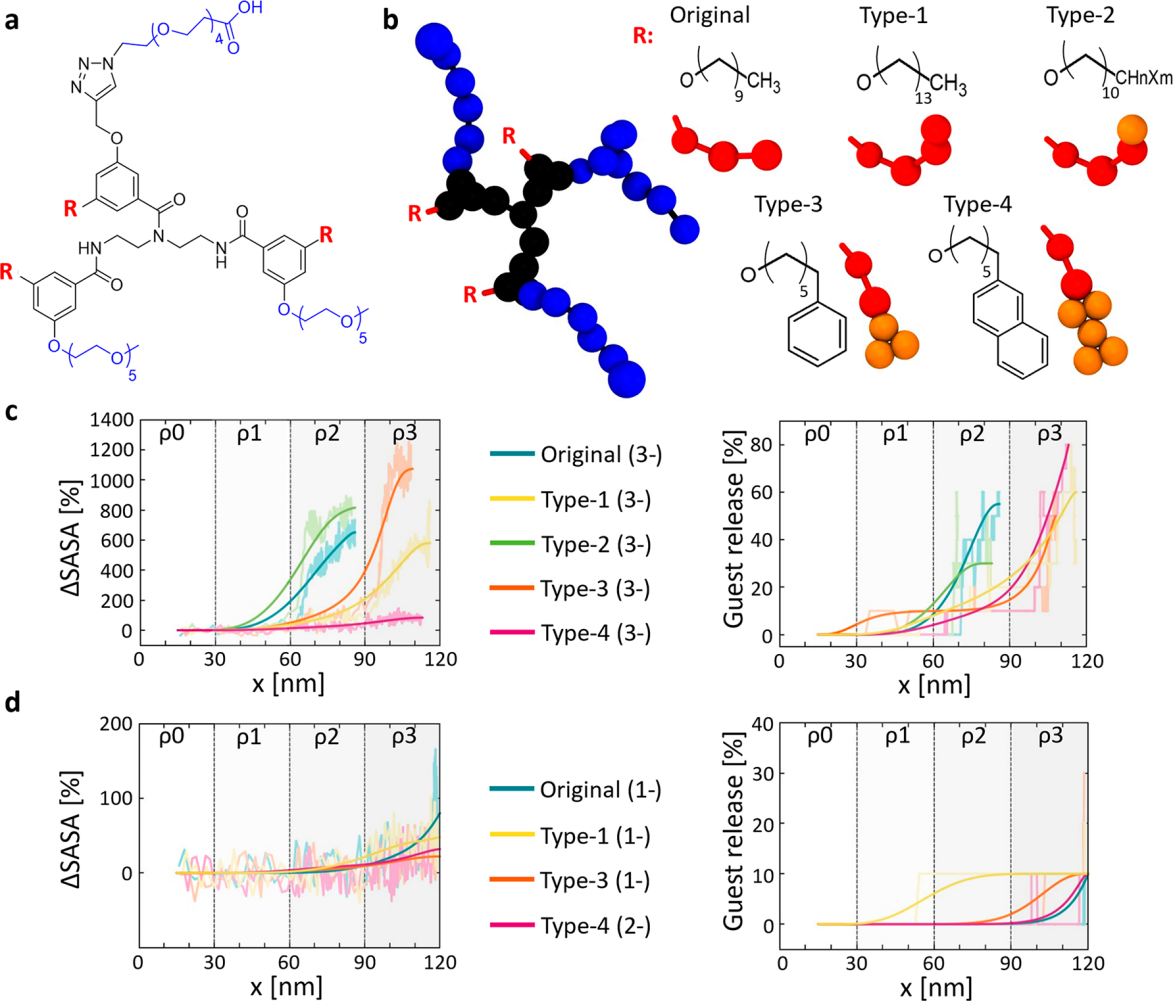
Modulating the NP chemotaxis and responsiveness by tuning the hydrophobicity of the self-assembling units. (a) Original reference oligomer (C10 hydrophobic tails). (b) Hydrophobic groups of the Type-1, Type-2, Type-3, and Type-4 oligomer variants and their corresponding fCG models. (c) Comparison of ΔSASA (NP SASA variation) and percentage of guest release for NPs composed of the different trivalent (−3*e*) oligomer variants. Same data for the (−2*e*) NP variants are reported in the Supporting Information (see Figures S8 and S9). (d) Similar NP behaviors can be obtained by NPs composed of oligomer variants having a similar Δ*E*_ass_/Δ*E*_bind_ balance. Raw data are shown in transparent colors, solid trend lines are shown to guide the eye.

The identification of a threshold receptor density as a function of multivalent interactions has been recently estimated by a sophisticated image postprocessing approach.^19^ However, perhaps the main advantage of these self-assembling oligomers is that their modular structure enables the fine-tuning of hydrophobic/hydrophilic groups in order to control the NP disassembly and the release of the guests during the NP chemotaxis. While the Δ*E*_ass_/Δ*E*_bind_ ratio is critical to control the chemotactic responsive behavior of the NPs, our simulations highlight how such a control can be achieved by for example, modulating the multivalent interactions between the NP and the surface, or even by changing the number of −1*e* charged groups on the oligomers (namely, changing the Δ*E*_ass_/Δ*E*_bind_ by acting on the Δ*E*_bind_). However, the Δ*E*_ass_/Δ*E*_bind_ ratio may be modified also by altering the hydrophobic groups in the oligomers. This has been recently done for similar self-assembling oligomers, showing some effects on the temperature-responsive behavior of the NPs that these form.^35^ As a further proof of concept, we thus studied the effect of changing the hydrophobic moieties in the oligomer units on the NP chemotaxis. Considering the reference oligomer of Figures 3 and 4 (named Original in Figure 6b), we systematically replaced its C10 hydrophobic units. Shown in Figure 6b, we obtained a Type-1 monomer variant by adding four carbon units (C14), that is, the equivalent of 1 hydrophobic CG bead in our fCG model. A Type-2 monomer variant carries a halogenated carbon group (orange) at the end of the Original structure, making the hydrophobic tails of the oligomers more hydrophilic compared to the Original saturated alkyl chains. Finally, we substituted the last CG bead in the decyl tails of the Original oligomer with phenyl and naphthyl functional groups, obtaining, respectively, Type-3 or Type-4 oligomer variants (Figure 6b). It is worth noting that the two last modifications affect not only the aggregation strength, but also the assembly shape, due to different packing interactions between the cyclic functional groups.

As a proof of concept, we repeated the *in silico* experiments of Figure 5, running non-equilibrium pulling CG-MD simulations for the Types 1–4 oligomers (carrying a variable amount of charges on the hydrophilic groups: −1*e*, −2*e*, −3*e*), and compared the behavior of these to the Original NPs (Figure 6c displays the comparison between the new NP variants compared to Original reference one). Since Type-1, Type-3, and Type-4 are more hydrophobic than the Original oligomer, their NPs are more stable. In Type-1 and Type-3 NP variants, the complete disassembly/exfoliation takes place only at density ρ_3_, where the multivalent interactions with the surface are stronger than in ρ_2_, where the Original −3*e* NP disassembles (Figure 6c, left, ΔSASA data). Again, we observe that the percentage of guest is consequent and follows the NP disassembly (Figure 6c, right). Interestingly, in Type-4 the monomer–monomer interaction is so strong that this NP variant deforms/reconfigures at higher ρ, and guest relase is observed at ρ_3_ even in the absence of complete NP disassembly (Δ*E*_ass_ and Δ*E*_bind_ are both strong and compete with each other). Similar data are reported for all −1*e* and the −2*e* NP variants in the Supporting Information (see Figures S9 and S10). These results clearly demonstrate that the strength of the NP-surface interaction is not the unique important factor, but the Δ*E*_ass_/Δ*E*_bind_ balance (thus controllable also by changing the Δ*E*_ass_) is the key parameter controlling the behavior of these NPs. To this regard, Figure 6d clearly shows how comparable behaviors in the system can be obtained with different NPs. While these cases change both in the hydrophobic groups and in the number of NP binding charges, evidently such combinations produce comparable global Δ*E*_ass_/Δ*E*_bind_ balances in the NPs, which make them behave on the surface in a similar way. It is interesting to note, for example, how Type-1 and Type-3 NPs composed of −1*e* charged oligomers do not disassemble and do not release guests during the CG-MD runs, behaving in the same way as a Type-4–2*e* charged NP. While the former NPs are less tightly assembled, the latter NP is more stable (stronger Δ*E*_ass_), thus requiring a stronger interaction with the surface (stronger Δ*E*_bind_) to behave in a comparable way. A comparative analysis of the kinetics of such fCG-NPs in regimes where these preserve their integrity (before severe deformation or disassembly) is provided in the Supporting Information. The velocity and the mean square displacement (MSD) of the fCG-NP computed from the MD simulations (Figure S11) show how the kinetics of the NP movement on the surface is affected (i) by the receptor density on the surface (ρ), (ii) by the charges present on the self-assembled oligomers (as these determine the Δ*E*_bind_), and ultimately also (iii) by the stability of the assembled NPs (Δ*E*_ass_). In particular, the data of Figure S11 indicate that the diffusion of the fCG-NPs becomes slower when the NPs visit higher ρ surface regions. In those areas, the Δ*E*_bind_ increases and the NP–surface adhesion strengthens (enhanced multivalent interactions) slowing down the diffusion of the NPs. Our evidence demonstrates that the behavior of the NP becomes consequently more and more subdiffusive as the receptor density on the surface increases, up to a limiting case: where the Δ*E*_bind_ becomes stronger than the Δ*E*_ass_. When the fCG-NP binds onto surface areas for which Δ*E*_bind_ > Δ*E*_ass_, it becomes more likely to observe the NP disassembling, rather than moving, under the influence of the external flux (diffusion 0 limit).

The perspective provided by these *in silico* investigations is quite neat and intriguing, as it suggests that once the density of receptors on a surface is known, it is in principle possible to rationally design the NP to control at what density this will disassemble and will release the encapsulated guests in the presence of an external flux (or at what density the NP would simply stop in the absence of any flux—see Figure 2 and Figure 4).

## Conclusion

In this work we have designed a concerted computational strategy to investigate supramolecular NPs with interesting chemotactic and density-responsive bioinspired properties. We have used a combination of multiscale molecular models and advanced simulation approaches to track, monitor, and ultimately to understand the dynamic behavior of the self-assembled NPs on receptor-grafted surfaces. Moving from minimalistic (mCG) to finer (fCG) models, we have uncovered the physical basis that controls the chemotactic behavior of the NP. First, we have unveiled the key role played by the competition between the self-assembly stability of the NP (Δ*E*_ass_ energy) and the strength of the interaction between the NP and the surface (Δ*E*_bind_ energy). Such Δ*E*_ass_*vs* Δ*E*_bind_ competition controls the autonomous motion of the NP along chemical gradients and also the fate of the NP. Second, using soft NPs made of modular self-assembling multivalent oligomers as an example case study, we provided chemical relevance to our results, showing chemical routes to achieve controlled bioinspired chemotaxis in artificial self-assembled systems. In fact, by tuning the NP surface charges and the chemical structure of the monomer units (*e.g.*, the nature of the hydrophobic groups in the self-assembling oligomers), it is possible in principle to control how the NP binds to the surface, its spontaneous diffusion on surface chemical gradients, its rolling, stopping, and, in the case of an external stimulus (*i.e*., a flux), even NP disassembly and guest-release in controlled spots of the surface. The results discussed herein suggest that such *in silico* experiments can be extremely valuable for augmenting our understanding of how to customize the structure of the self-assembling units to control the stability of the NP, the Δ*E*_ass_/Δ*E*_bind_ balance, and the dynamic behavior of these chemotactic NPs. Furthermore, we show that it is in principle possible, once the features of a target surface are known, to rationally design or customize *ad hoc* NPs in order to achieve controllable chemotaxis in artificial molecular systems.

## Materials and Methods

All simulations were conducted using GROMACS 2018.6^47,48^ patched with PLUMED 2.5.^49^ The VMD visualization suite was used to display and render the simulated systems.

### Minimalistic Coarse-Grained (mCG) Model and Simulations

The minimalistic mCG model is characterized by three different bead types representing (i) the monomers within the assembly, (ii) the guest particles contained inside the self-assembled NP, and (iii) the receptors grafted on the surface. The interactions have been defined using a Lennard-Jones (LJ) 12–6 potential, initially setting the LJ parameters to σ = 0.47 nm and ε = 10 kJ mol^–1^, for the monomer–monomer interactions (Δ*E*_ass_) and to σ = 0.35 nm and ε = 40 kJ mol^–1^ for the monomer–receptor interaction (Δ*E*_bind_). This provided a Δ*E*_ass_/Δ*E*_bind_ ratio of ∼1/4 comparable to that recently estimated in the case of similar self-assembling oligomers containing a ligand capable of specifically bind to a complementary receptor protein (based on the avidin–biotin interaction).^13^ We also added on the surface a weakly 9–3 LJ attractive potential of 2.0 kJ mol^–1^ (considerably weaker than specific interactions), in order to mimic the weak nonspecific interactions between the NP and the surface and to prevent the NP from penetrating inside the surface in receptor-free surface regions. In this simplified mCG model, the surface receptor CG beads were kept frozen during the simulations. The NP–surface system was initially minimized using a steepest descent algorithm, and a leapfrog stochastic dynamics integrator was used for the production run. A Langevin dynamics was conducted using Coulomb and van der Waals cutoffs of 1.1 nm, and a relative dielectric constant of ε_r_ = 15 (to implement electrostatic screening of the solvent, accordingly with the Dry MARTINI force field standards).^39^ All simulations using this model have been conducted at 300 K of temperature, in NVT conditions (constant *N*, number of particles; *V*, volume; *T*, temperature) using a 20 fs time step. Because of the anisotropic nature of the system, periodic boundary conditions were considered only along *x*- and *y*-axis. Metadynamics (MetaD) simulations were used to enhance the NP sampling of the surface. A MetaD bias was applied on the *x*- and *y*-distances (used as the collective variable, CV) of the NP center of mass respect to the origin of the system, depositing every 5000 steps Gaussian kernels of height 20 kJ/mol^–1^ and width of 1.0 for both variables.

### Submolecular Resolution Models (fCG) and Simulations

#### AA and Fine fCG Models

The atomistic model was built with Avogadro^50^ based on the chemical structure of the oligomers. The oligomers were created as composed of three main parts (hydrophobic tails, hydrophilic tails, and core), which have been parametrized based on the General AMBER Force Field (GAFF),^51^ using Antechamber.^52^ The fine fCG models of the oligomers were built based on the MARTINI force field.^38^ The bonded force field parameters of the fCG models have been optimized automatically to reproduce the bond, angle, and dihedral distributions of the AA-MD simulations using Swarm-CG.^40^ The nonbonded parameters have been optimized by choosing the appropriate MARTINI bead types in order (i) to reproduce the radius of gyration and the solvent accessible surface area (SASA) of the all-atom model seen in AA-MD simulations and (ii) to reproduce the free-energy of dimerization between two oligomers in water obtained *via* metadynamics (AA-MetaD *vs* CG-MetaD) simulations between two monomers (see Figure S5 in the Supporting Information). For the best reliability, first a wet MARTINI-based CG model was created and optimized, which was then used to optimize a Dry MARTINI-based CG model, fCG, which has been then used for the simulations of Figures 3–6. The self-assembled NPs were obtained by inserting a large number of fCGmonomers in a box and a classical MD simulation was run. A largeand stable NP model was obtained *via* self-assembly of44 fCG monomers. This fCG NP model was then used as a reference in all the simulations. Ten CG beads were also added inside the aggregate in order to represent guest particles. The interactions of such guest particles are weak enough to allow the prompt monitoring of their release in case of the NP’s disassembly (LJ parameters: σ = 0.43 nm and ε = 6.5 kJ mol^–1^). The alkylamine groups on the surface were also modeled at the same resolution level, based on the Dry MARTINI force field. In detail, the amino-groups are defined by three CG beads: a base one, grafted to the surface, a central CG bead (mimicking a carbon linker), and a charged hydrophilic head. To keep the receptor position fixed, the base CG beads of the receptor groups were kept frozen during the simulations. Complete structures and parameters for all CG models used here-in are available at https://doi.org/10.5281/zenodo.5517760 (or at https://github.com/GMPavanLab/RollingNP).

### Unbiased Simulations

Since all the simulations were performed in implicit solvent, the relative dielectric constant was set to ε_r_ = 15 to model the electrostatic screening of the solvent (standard for the Dry MARTINI force field).^39^ In addition, explicit counterions were added to neutralize the systems charge. All CG-MD simulations of the fCG model were run in NVT conditions (constant *N*, number of particles; *V*, volume; *T*, temperature) at *T* = 300 K. All the systems were preliminarily minimized using a steepest descent algorithm, and a leapfrog stochastic dynamics integrator was then used for all unbiased MD production runs, using a 20 fs time step, and Coulomb and van der Waals cutoffs of 1.1 nm. For the CG-MD simulations of the static NP adhesion on different receptor density regions (Figure 3d), the different NP models were initially centered on top of four different 20 × 20 nm^2^ surfaces characterized by four different densities: ρ_1_ = 0.034 rec/nm^2^, ρ_2_ = 0.09 rec/nm^2^, ρ_3_ = 0.64 rec/nm^2^, and ρ_4_ = 1 rec/nm^2^, while each system was then equilibrated for 1 μs of CG-MD simulation.

### Infrequent MetaD Simulations for the Study of NP Unbinding/Escape

We ran 30 infrequent CG well-tempered MetaD simulations to obtain information on the characteristic time scale and the associated free energy barrier that has to be crossed in the system to detach a NP (represented with the fCG model and composed of −1*e* self-assembled oligomers) from a single bound surface receptor (Figure 4a: time scale for breaking a monovalent/single NP–receptor interaction). In these runs, we used as the CV the number of contacts between the NP’s charged beads and the surface receptor CG beads. We used a bias factor of 10, a Gaussian height of 1.2 kJ mol^–1^, a deposition stride of 1 Gaussian every 50 000 time steps with a sigma of 0.5 nm. Simulations were terminated once the number of contacts dropped to 0. The characteristic time scale for the NP unbinding event was calculated from the poissonian fit of the unbiased transition times distributions obtained from the 30 infrequent MetaD runs. The unbiased transition time (*t*) can be calculated from each individual MetaD run as:
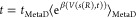
where *V*(*s*(*R*),*t*) is the time dependent bias provided for the transition during the run, the exponential (brackets) is averaged over the MetaD run and β is *kT*^–1^. The transition times (*t*) calculated from the MetaD runs were then used to build the transition probability distribution *P*_*n*≥1_ (namely, the probability to observe at least one NP unbinding event by time *t*:
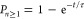
where τ is the characteristic time for rare NP unbinding event. Figure S8 shows the exchange times collected from the individual runs. These fit well with a poissonian transition probability distribution *P*_*n*≥1_, demonstrating the appropriateness of the used setup. From the *P*_*n*≥1_ distribution, it is possible to calculate the characteristic time scale (TAU: τ) for the NP unbinding from a single/monovalent interaction with a surface receptor (see Figure 4a and Figure S8).

### Multiple-Walker CG-MetaD Simulations

In the multiple-walker MetaD simulations, a surface model of 60 × 60 nm^2^ was built as composed of different receptor density areas (see Figure 4). Multiple-walker MetaD was used to run in parallel 36 simulations of the same fCG system. The bias acted along the *x*- and *y*-distance of the NP’s center of mass from to the origin of the system. The bias was constructed by depositing every 500 CG-MD steps Gaussian kernels of height 1.2 kJ mol^–1^ and width of 0.1 for both variables. Repeating the simulations using or not-using the Particle Mesh Ewald (PME) summation to treat long-range electrostatics provided consistent results, proving the general validity of the approach in exploring the chemotactic NP behavior on the surface.

### *In silico* NP Rolling and Exfoliation Experiments

We built a surface model having size 120 × 30 nm^2^, characterized by four consecutive receptor density regions: ρ_0_ = 0 rec/nm^2^, ρ_1_ = 0.12 rec/nm^2^, ρ_2_ = 0.52 rec/nm^2^, and ρ_3_ = 1.12 rec/nm^2^, 30 × 30 nm^2^ each (see Figure 5a). In these CG-MD simulations, we used the same NPs composed of 44 assembled oligomers used in Figures 2 and 3, namely our fCG model of NP. The CG-MD runs were conducted in NVT conditions at the temperature of 300 K, while Coulomb and van der Waals interactions were modeled using a 1.1 nm cutoff. During these MD runs, a constant force F = −100 kJ mol^–1^ nm^–1^ was applied on the center of mass of the NP, directed along the main *x*-axis of the surface (Figure 5a), in order to obtain a pulling effect on the NP comparable to that of a flux similar to that existing on the blood vessels (NP diffusion rate: ∼0.5–1 cm/s).^46^ To avoid NP jumping far from the surface in the (receptor-free) region, a wall on the *z*-axis was added using the PLUMED plugin on the center of mass of the NP at 5 nm with kappa = 150.0 and exp = 2.

## Supporting Information Available

Additional details on the structural illumination microscopy (SIM) and on the molecular models, additional materials, data and figures from the molecular simulations (PDF)
MetaD simulation of mCG-NP (MPG) (MPG)
MetaD simulation of fCG-NP (MPG) (MPG)
Flux of fCG-NPs (MPG, MPG, MPG)

## Supplementary Material

nn1c05000_si_001.pdf

nn1c05000_si_002.mpg

nn1c05000_si_003.mpg

nn1c05000_si_004.mpg

nn1c05000_si_005.mpg

nn1c05000_si_006.mpg

## Notes

Complete details of all molecular models used for the simulations, and of the simulation parameters (input files, *etc*.) are available at https://doi.org/10.5281/zenodo.5517760 (or at https://github.com/GMPavanLab/RollingNP).
