# Supplementary material for: Toward Chemotactic Supramolecular Nanoparticles: From Autonomous Surface Motion Following Specific Chemical Gradients to Multivalency-Controlled Disassembly
Source: ACS Nano. 2021 Sep 22;15(10):16149–61. doi: 10.1021/acsnano.1c05000 (PMC8552489; doi:10.1021/acsnano.1c05000)
Supplement: Supplementary file 1 — nn1c05000_si_001.pdf [file nn1c05000_si_001.pdf]

Supporting Information for:

Toward Chemotactic Supramolecular Nanoparticles: From Autonomous Surface Motion Following Specific Chemical Gradients to Multivalency-Controlled Disassembly

Chiara Lionello,[†,a] *Andrea Gardin,*[†,a] *Annalisa Cardellini,*[a] *Davide Bochicchio,*[b,c] *Manisha Shivrayan,*[d] *Ann Fernandez,*[d] *S. Thayumanavan*[d] *& Giovanni M. Pavan*[a,b,*]

[a] Department of Applied Science and Technology, Politecnico di Torino, Corso Duca degli Abruzzi 24, 10129 Torino, Italy

giovanni.pavan@polito.it

[b] Department of Innovative Technologies, University of Applied Sciences and Arts of Southern Switzerland, Polo Universitario Lugano, Campus Est, Via la Santa 1, 6962 Lugano-Viganello, Switzerland

[c] Department of Physics, Università degli studi di Genova, Via Dodecaneso 33, 16100 Genova, Italy

[d] Department of Chemistry, Center for Bioactive Delivery at the Institute for Applied Life Sciences, University of Massachusetts, Amherst, Massachusetts 01003, United States

Contents

Experiments 2
Structural Illumination Microscopy (SIM) 2
Modelling Details 5
Molecular Dynamics (MD) of Minimalistic Coarse-Grained (CG) mCG-NP model 5
Molecular Simulations of Fine Coarse-Grained Nanoparticle fCG-NP models 6
Infrequent Metadynamics (MetaD) Simulations 8

Experiments

Structural Illumination Microscopy (SIM)

The quantification and rearrangement of trimeric assemblies on positively charged polylysine surfaces was carried out *via* Structural Illumination Microscopy (SIM). First, 1 mg of either 1, 2 or 3 COOH trimer was dissolved in 100 µL acetone. To this, a 1 mg/mL solution of DiD (1,1'-Dioctadecyl-3,3,3',3'-Tetramethylindodicarbocyanine, 4-Chlorobenzenesulfonate Salt) was loaded at 10 v/v%. 1 mL DI water was then added dropwise and stirred overnight to obtain trimeric assemblies dispersed in water and non-covalently encapsulating DiD dye. Solutions of the trimeric assemblies were then diluted to 0.05 mg/mL for SIM experiments. For experiments with SIM, a glass chamber setup was fabricated. Briefly, two pieces of clear double-sided tape of dimensions 1 cm x 3cm x 70 µm were placed on the polylysine functionalized glass slide 1 cm apart. A coverslip was then placed over the double-sided tape to form a thin chamber. For pH studies with SIM, 20 µL of the solution containing trimeric assemblies were injected into the glass chamber. For studies at neutral pH, 20 µL phosphate buffer at pH 7 was injected into the chamber and allowed to sit for 2 minutes. This was followed by a washing step to remove the buffer by injecting 75 µL DI water. Similarly, for studies at low pH, 20 µL phosphate buffer at pH 4 was injected into the chamber and allowed to sit for 2 minutes. This was followed by the washing step to remove the buffer by injecting 75 µL DI water. Images were then captured, and the average number of particles counted using Nikon-NIS Elements.

In Figure S1, the fluorescent dots in the SIM experiments refer to the number of NPs bound to the surface. Figure S1 shows that at neutral pH (pH = 7), because of the deprotonation of the COOH groups (into COO^- groups), the number of NPs bound to the surface increases with the number of charged COO^- terminal groups in the oligomer (increased multivalent binding interactions). This is quantified in the histograms in Figure S2. These experimental data are in agreement with the trends obtained from the fCG models in Figure S6, where the number of contacts between fCG-NPs and receptors on the surface model increases with the oligomer charge, at all explored receptor densities. The selective electrostatic nature of the binding is also experimentally proven by the pH-dependent experiments (see Figure S1 going from neutral (a) to low pH (b)) the level of deprotonation and the number of COO^- groups strongly decreases, reducing the strength of electrostatic binding and consequently the number of particles bound on the surface.

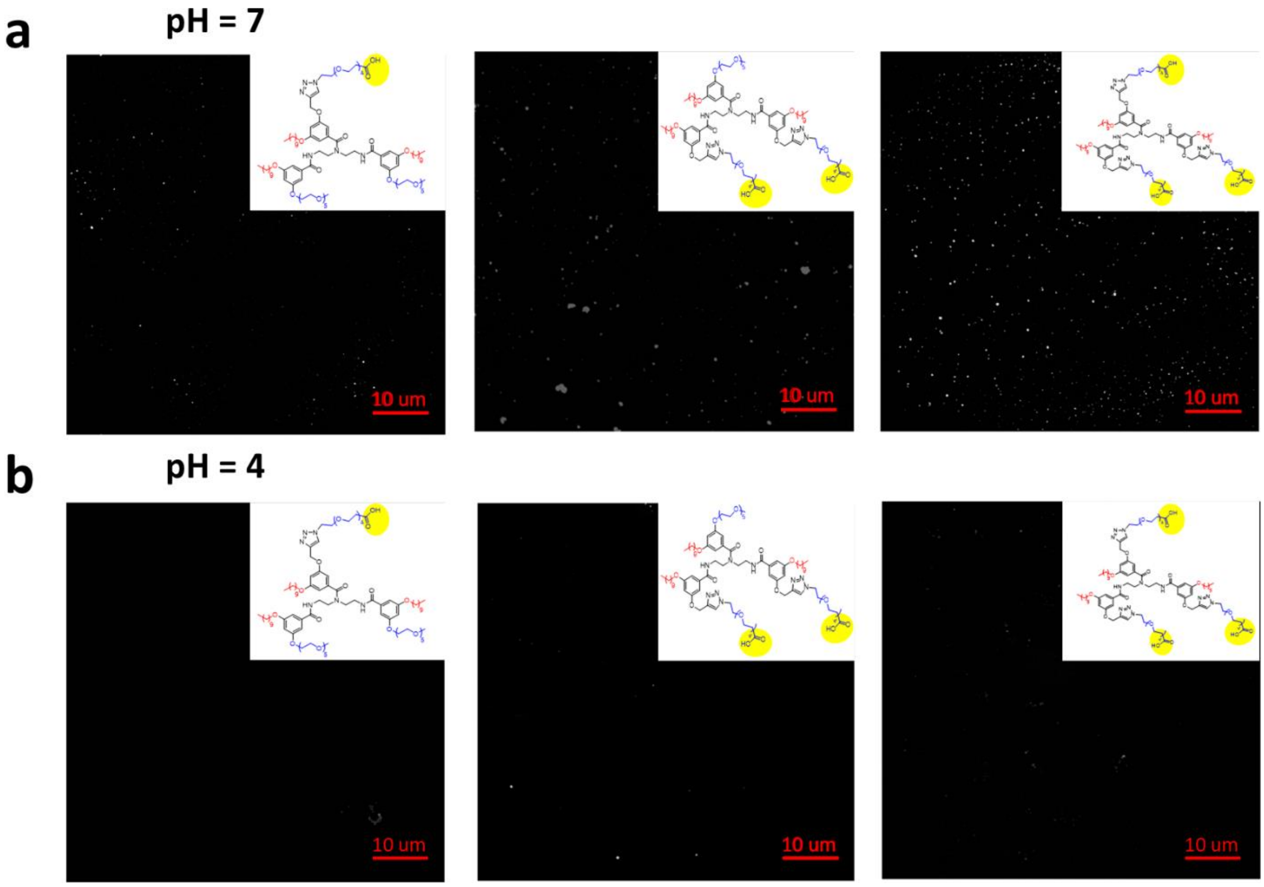

Figure S1: Structural Illumination Microscopy (SIM) images of a polylysine functionalized surface showing adhesion of 1-COOH, 2-COOH, 3-COOH trimeric assemblies at pH = 7 (a) and pH = 4 (b). White dots identify the surface bound NPs.

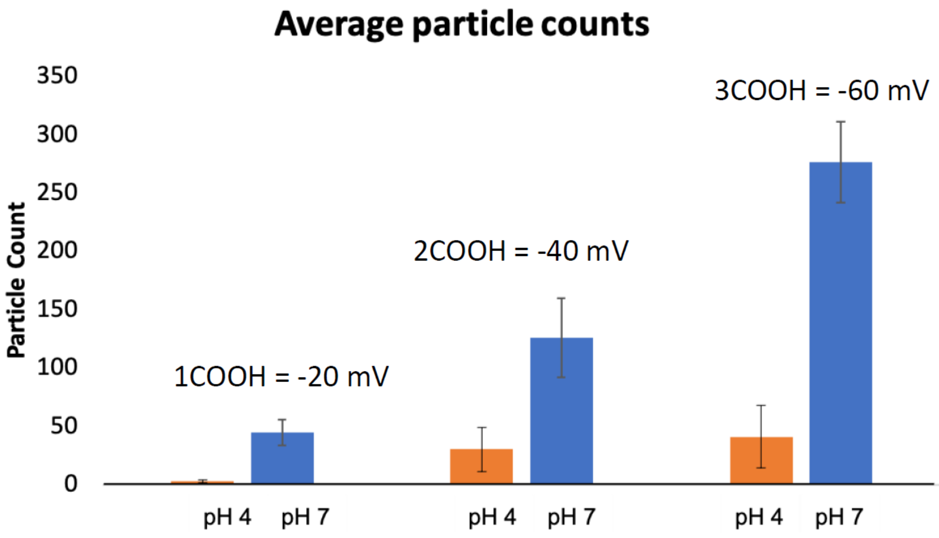

Figure S2: Quantification of trimeric assemblies on polylysine surfaces using SIM at pH = 7 and pH = 4. Increasing the multivalent interaction, the average particles adhered to the surface rise up in case of neutral pH, instead the average particle counts remain constant in case of pH = 4, remarking the increase of protonation of COO^- terminal groups.

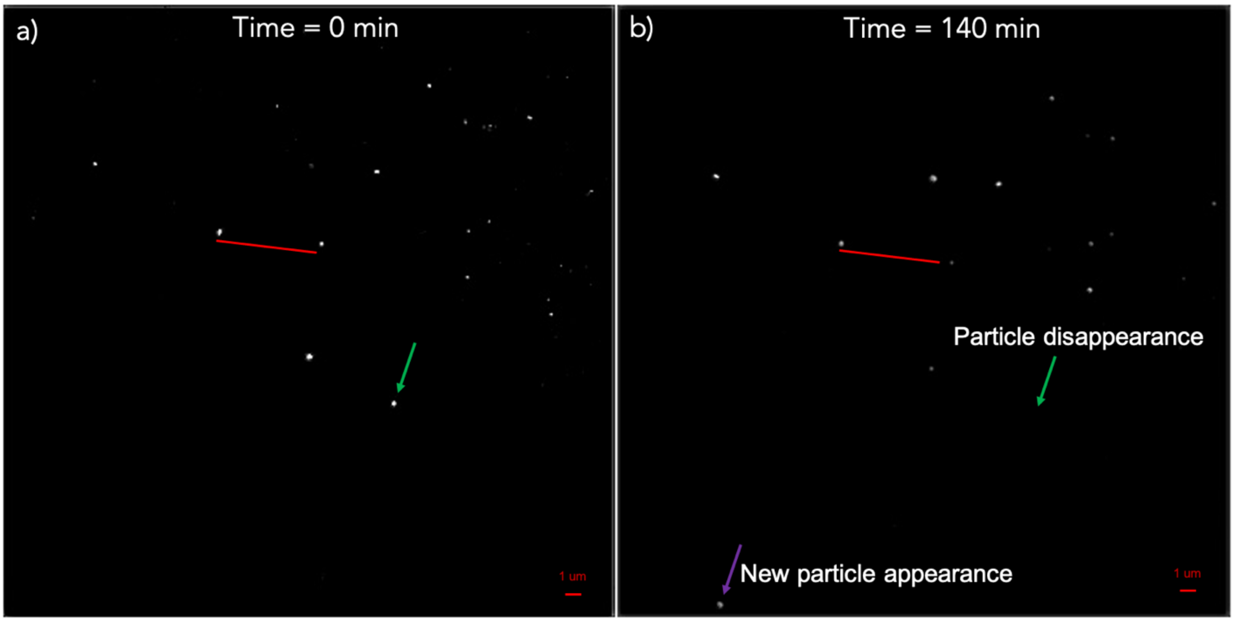

Figure S3: Structural Illumination Microscopy (SIM) images of a polylysine functionalized surface showing a) adhesion of 1-COOH trimeric assemblies at the start of the experiment and b) rearrangement of the trimeric assemblies as captured by the microscope after 140 minutes.

Modelling Details

Molecular Dynamics (MD) of Minimalistic Coarse-Grained (CG) mCG-NP model

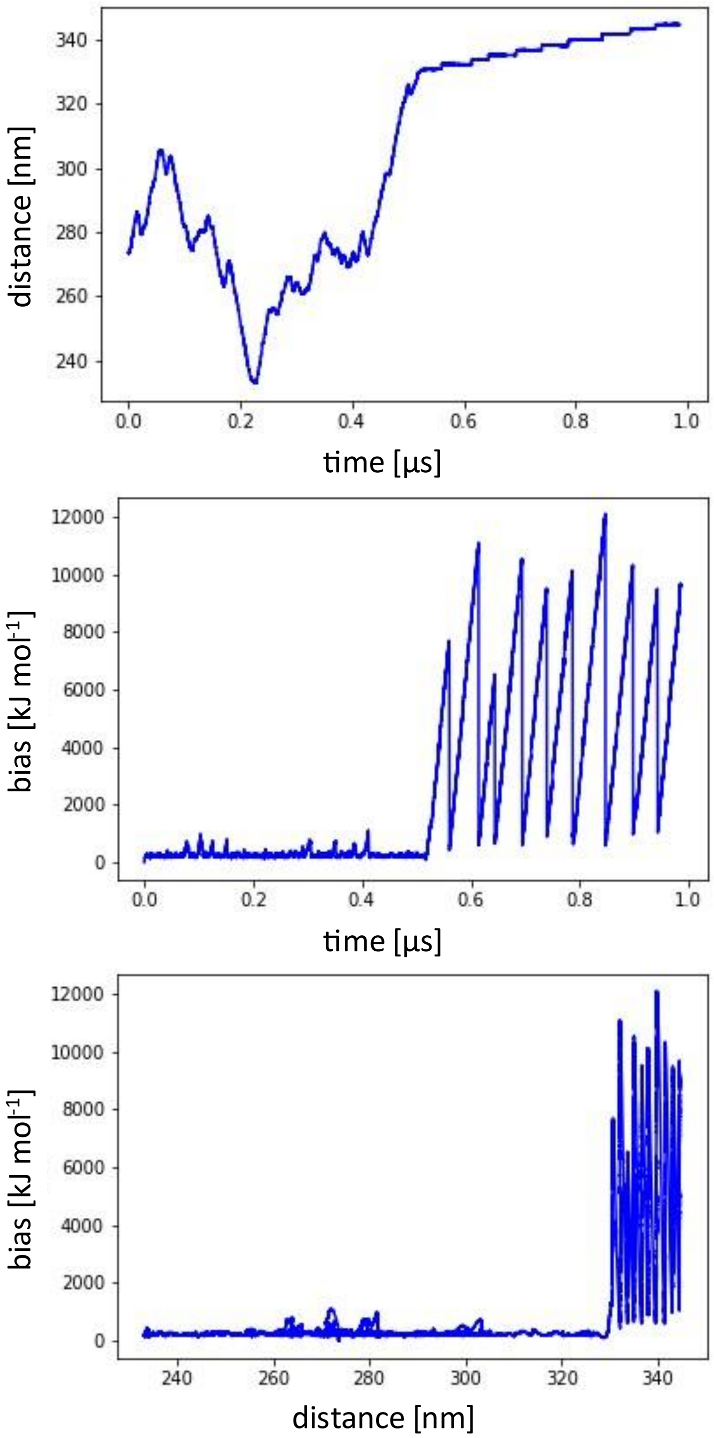

***Figure S4**:* Metadynamics (MetaD) simulation details of the minimalistic coarse-grained (CG) supramolecular NP with encapsulated guests rolling on a receptor decorated surface. Top panel: time evolution of NP vector position. Central panel: time evolution of accumulated bias. Note that once the NP binds to the higher density surface (at time = 0.5 µs), the bias accumulated increases rapidly. Bottom panel: accumulated bias as function of NP vector position during the MetaD simulation.

Molecular Simulations of Fine Coarse-Grained Nanoparticle fCG-NP models

Well-Tempered MetaDynamics (MetaD) simulations were used to validate the dimerization Free Energy Surface (FES) profile of the Coarse-Grained (CG) model of two trimeric amphiphiles with respect to the FES profile of the all-atom (AA) model of the same dendrimer (Figure S1). Specifically, we identified two atomistic collective variables: (i) the distance between the central residues within the monomers, and (ii) the mean number of contacts between the hydrophobic residues (rational switch function and R_0 = 0.5). The CG collective variables were instead the distance between the central beads (corresponding to the central AA residue) and the mean number of contacts between the hydrophobic beads (treated in the same way as the atomistic). The two FES profiles were ultimately obtained by a reweight on the "central" distance. In both cases the simulation sofware PLUMED was used and the following simulation parameters were set: height = 2.0, biasfactor = 20, sigma = 0.05 and 0.1 for the variable distance and number of contacts respectively.

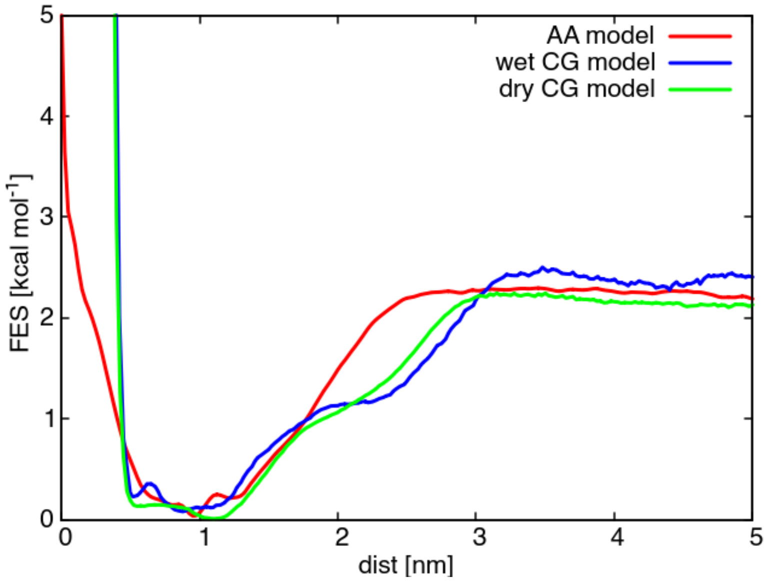

Figure S5. Free Energy Surface (FES) of trimeric amphiphile dimerization obtained with well-tempered metadynamics technique. The validation of Coarse-Grained (CG) model (blue line) has been tuned on the all-atom (AA) profile (red line) as a function of the distance between the center of the two monomers (see Figure 3a of the main paper). The distance variable refers to the COM distance (AA case) and the bead-to-bead distance (CG case) of the central residue of the standard monomer.

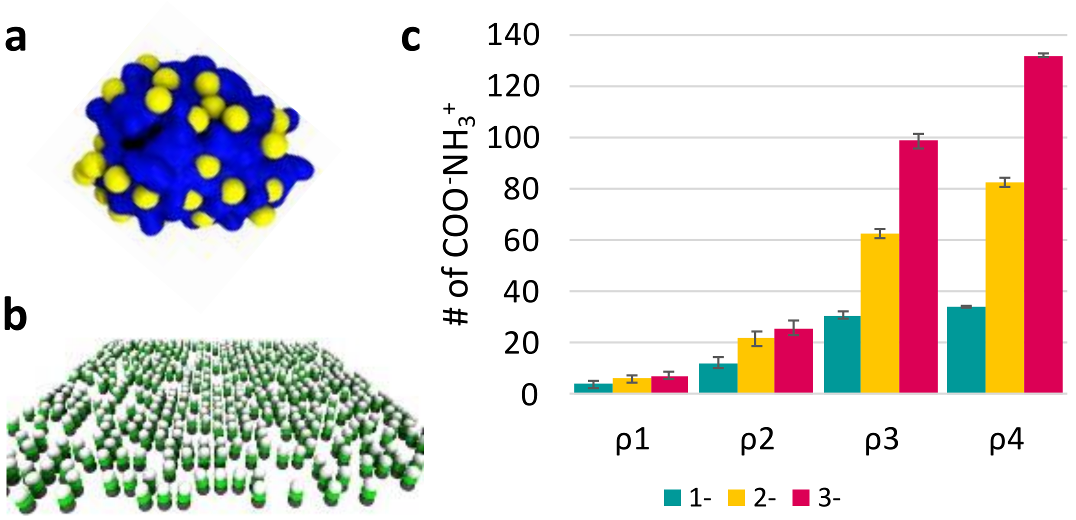

Figure S6. (a) CG-MD simulation snapshot of a single self-assembled nanoparticle made of 44 trimeric amphiphiles validated as shown in Figure S1. Color code: charged beads are depicted in yellow, while blue beads represent the complementary monomer structure. (b) An example of ligands decorated surface. The charged beads are in white, while the remaining ligands are colored in green and gray. (c) Number of contacts between the NP charged beads and the surface ligand charged beads calculated in unbiased CG-MD simulations for different ligand concentrations (ρ_1, ρ_2, ρ_3, ρ_4) and protonation states (-1*e*, -2*e*, -3*e*).

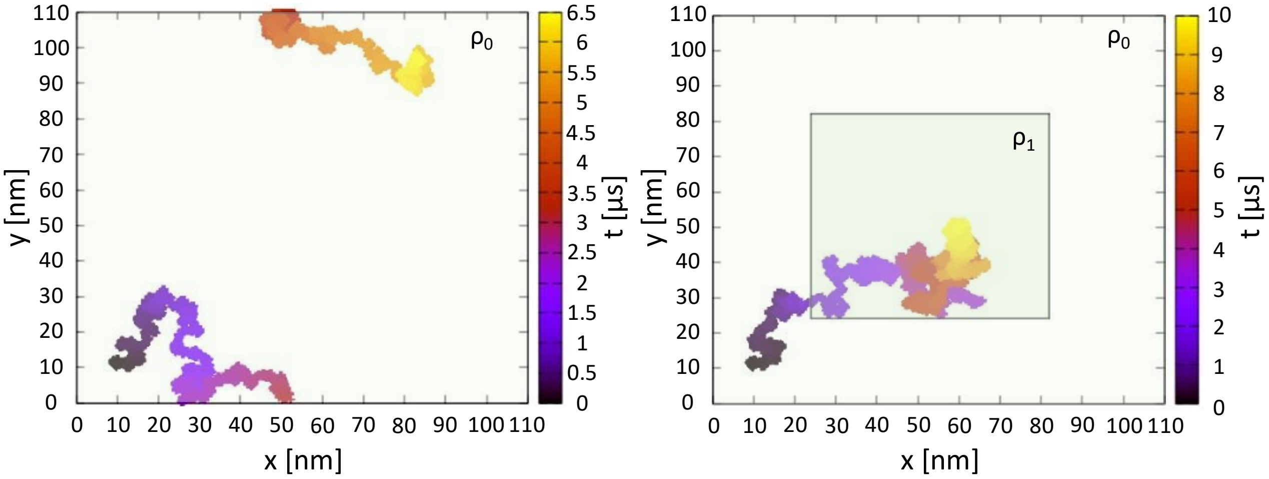

Figure S7: CG-MetaD trajectories of fCG-NPs onto different surface models. Left: The fCG-NP moves randomly in 2D in the case where the density of surface receptor groups is low and uniform (ρ_0). Right: Motion of the NP onto a surface with two different, yet relatively low, receptor density regions (ρ_0 and a higher ρ_1). The NP motion becomes irreversible when it visits regions where the ρ is high enough to have $\Delta E_{bind} > \Delta E_{ass}$.

Infrequent Metadynamics (MetaD) Simulations

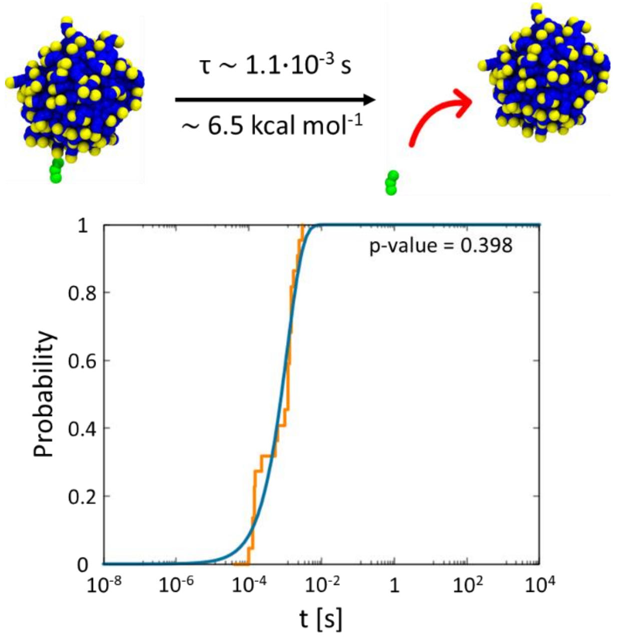

Figure S8: Transition times extracted from the infrequent MetaD simulations. The plot reports the transition times collected from the replica infrequent MetaD simulations (orange segments), and the related Poissonian fitted distribution (in blue), for the detachment of the -1*e* charged fCG-NP from a single surface ligand (monovalent interaction)***.*** The analysis provides an estimated unbinding timescale of $\boldsymbol{\tau = 1.14 \cdot 10^{-3}\ s}$.

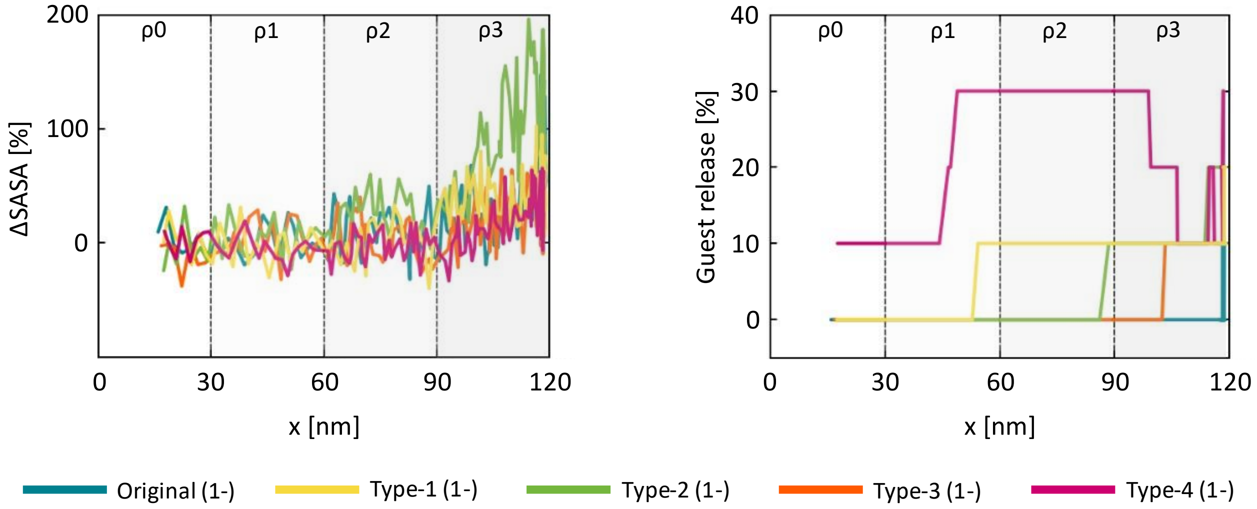

Figure S9: Comparison of ΔSASA (NP SASA variation) and percentage of guest release for NPs composed of the different trivalent (-1*e*) dendron variants. The CG models of *Original*, *Type-1*, *Type-2*, *Type-3* and *Type-4* residues are reported in Figure 6 of the main paper.

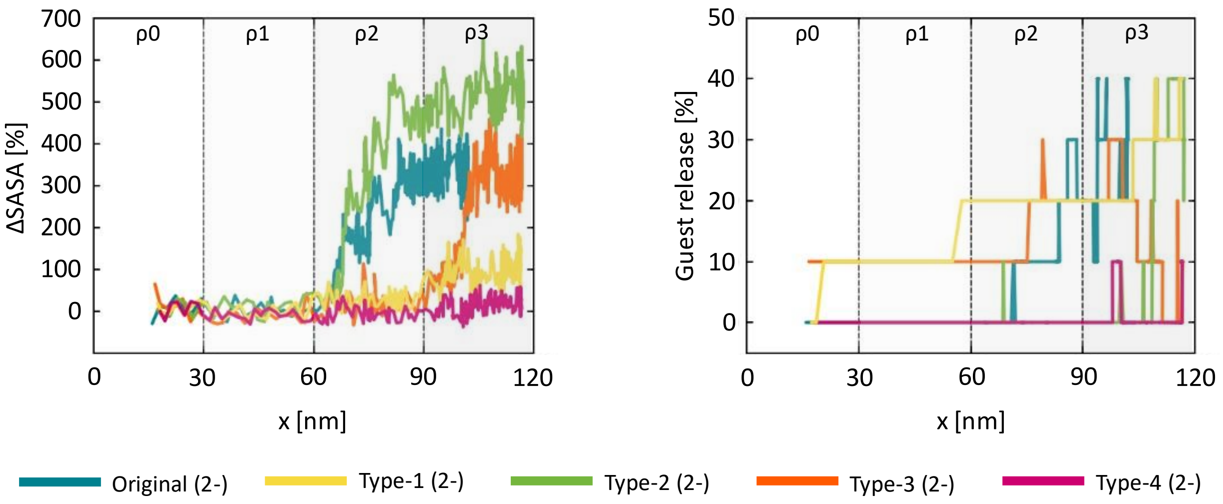

Figure S10: Comparison of ΔSASA (NP SASA variation) and percentage of guest release for NPs composed of the different trivalent (-2*e*) dendron variants. The CG models of *Original, Type-1, Type-2, Type-3* and *Type-4* residues are reported in Figure 6 of the main paper.

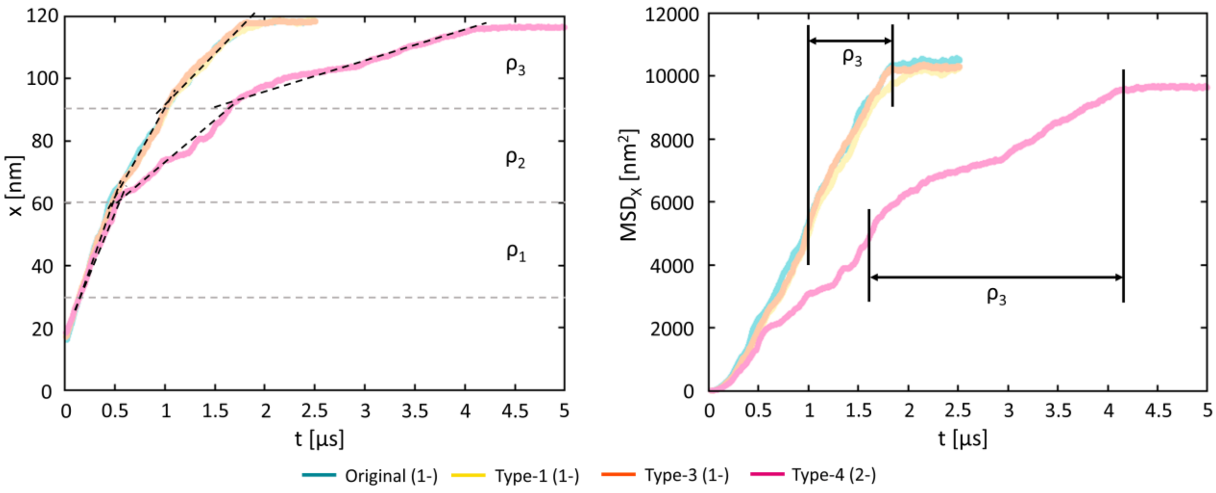

Figure S11: X-position (x(t)) and x-Mean Square Displacement (x-MSD) of the fCG-NPs (*Original*, *Type-1* (-1*e*), *Type-3* (-1*e*), and *Type-4* (-2*e*)) along the receptor-density-gradient surface displayed in Figure 5a of the main manuscript. The slope of the x(t) profiles represents the fCG-NP velocity along x direction. The data show that the higher is the receptor density on the surface (ρ), or the oligomer's charge (colored curves), the lower is the slope of the x(t) (black dotted lines): *i.e.*, the fCG-NP motion on the surface becomes slower while the multivalent interactions between the NPs and the surface increase.
